# Dynamic force-induced nucleoporin stretch mechanomemory prolongs nuclear pore complex dilation and importin transport

**DOI:** 10.1063/5.0345809

**Published:** 2026-11-04

**Authors:** Fazlur Rashid, Sadia Amin Kabbo, Ning Wang

**Affiliations:** 1 The Institute for Mechanobiology, Northeastern University, Boston, Massachusetts 02115, USA; 2 Department of Bioengineering, College of Engineering, Northeastern University, Boston, Massachusetts 02115, USA; 3 Department of Mechanical Science and Engineering, The Grainger College of Engineering, University of Illinois at Urbana-Champaign, Urbana, Illinois 61801, USA

## Abstract

How the nuclear pore complex (NPC) is dynamically regulated by mechanical stimuli is poorly understood. Herein, using FRET (fluorescence resonance energy transfer) spectroscopy and MINFLUX (MINimal fluorescence photon FLUXes) nanoscopy, we demonstrate that external local mechanical and global osmotic stresses to the surface of mammalian cells induced mechanomemory of nucleoporin (NUP) deformation. During and long after (∼30 min) local stress application via integrins or global hypo-osmotic stress, the FRET ratio of YFP-NUP62 (acceptor) to CFP-NUP88 (donor) decreased and remained low, suggesting that the distance between the two NUPs increased and the NUPs were stretched. Independent MINFLUX measurements of NPC strain memory following the same local mechanical or global osmotic stress mirrored the magnitude of NUP strain memory in the NPC observed via FRET. This cross-method validation supports the interpretation that the decreased FRET ratio primarily represents NUP stretching. Compared with stiff elastic and slow-relaxing viscoelastic substrates, soft elastic and fast-relaxing viscoelastic substrates extended the duration of mechanomemory of nucleoporin deformation to 60 min after stress cessation. Mechanomemory of NPC dilation prolonged translocation of importins α and β1 through the NPCs and translocation of importin β1-dependent histone deacetylase 3 into the nucleus. Our findings reveal the structural basis of dynamic force-induced nucleoporin mechanomemory in controlling NPC diameter. This, in turn, regulates nucleocytoplasmic transport-mediated mechanotransduction in living cells.

## INTRODUCTION

It is known that mechanotransduction, the process by which mechanical stimuli are converted into biochemical signals and biological responses, is critical for the regulation of physiological homeostasis and disease.^1–3^ It is suggested that the nuclear pore complex (NPC), the dominant gateway, and LINC (linker of cytoskeleton and nucleoskeleton) play a vital role in nuclear mechanotransduction.^4,5^ Increasing evidence also suggests that the nuclear envelope and NPCs are not merely a passive conduit but can sense and respond to mechanical forces and facilitate nucleocytoplasmic transport.^6–9^ However, the mechanisms of mechanosensing by NPCs are still largely unknown. Various models have been proposed to explain how NPCs might regulate nucleocytoplasmic transport.^10^ Recent theoretical models showed that NPC dilation and cargo interaction with phenylalanine and glycine motifs (FG) rich NUPs (FG-Nups) exponentially enhance molecular transport rate.^11,12^ Experimental studies demonstrated that forces regulate nucleocytoplasmic transport, establishing a functional link between mechanical stimuli and transport via NPC.^7,13,14^ Additionally, studies further provided evidence that NPCs undergo force-dependent remodeling, suggesting that mechanical forces can directly alter NPC architecture.^15^ Tremendous progress has been made in recent years in detailing the molecular composition and structure of NPCs.^16–20^ Using CryoET (Cryo-Electron Tomography), researchers have shown that in fission yeast cells, NPCs of growing cells are in a dilated conformation, whereas under conditions of cellular energy depletion or hyperosmotic stress, they are in a constricted conformation.^17^ CryoEM (Cryo-Electron Microscopy) has been employed to visualize *X. laevis* nucleoporins (NUPs) in the NPC of isolated nuclear envelopes.^19^ Super-resolved MINFLUX (MINimal fluorescence photon FLUXes) nanoscopy^21,22^ has been employed to visualize NUPs in NPCs in permeabilized cells.^23^ Therefore, direct visualization of NPC dilation has been reported using CryoET, CryoEM, and MINFLUX. However, despite the progress in NPC's detailed structure and composition, how NPC diameter is regulated dynamically by mechanical stress to permit the selective passage of molecules remains elusive in intact living cells.

Over the last decade, it has been shown that living cells exhibit behaviors of mechanomemory.^24,25^ Mechanomemory is the biological response long after withdrawal of mechanical perturbation. Many studies on mechanomemory are focused on switching living cells from stiff to soft elastic substrates or vice versa, following being on the original substrates for days to weeks.^26–34^ Recently, we have studied the mechanisms of mechanomemory by studying early mechanotransduction events. We have demonstrated that short durations (2–10 min) of mechanical stimulation via integrins induce chromatin stretching dependent nuclear mechanomemory (lasting for ∼30 min).^35,36^ These short durations of stress elevate chromatin and nucleoplasm diffusivity, RNA polymerase II activity, and gene transcription after cessation of the applied stress.^35,36^ The extent of nuclear mechanomemory after cessation of applied stress on the cell surface appears to be associated with the state of NPC size.^35^

Hence, in this study, using fluorescence resonance energy transfer (FRET) spectroscopy and single- or two-color MINFLUX, we demonstrate that externally applied physiologically relevant local and global stresses to the surface of living intact cells induce mechanomemory of deformation of nucleoporins in the NPCs. We show that when a local rotational stress or a global hypo-osmotic stress is applied, nucleoporins are stretched and remain stretched after stress release. Applied stress leads to dynamic NPC diameter expansion and dilation memory in intact living cells. Soft elastic and fast-relaxing viscoelastic substrates extend the duration of mechanomemory of nucleoporin deformation when compared with stiff elastic or slow-relaxing viscoelastic substrates after cessation of stress. The mechanomemory of NPC dilation prolongs the translocation of single-molecule importin α and importin β1 through the NPCs. This NPC mechanomemory induces importin β1-dependent Histone Deacetylase 3 (HDAC3) nuclear translocation in living cells.

## RESULTS

### Nucleoporins exhibit dynamic deformation in response to transient stresses

To determine if and how NUPs in NPCs respond to external mechanical stresses, we applied local stresses on the cell surface via integrins using an RGD (arginine–glycine–aspartic acid)-coated magnetic bead (∼4 *μ*m in diameter) with the MTC (magnetic twisting cytometry)^37^ [Fig. 1(a)]. The magnetic bead was magnetized in the Y-direction with a strong magnetic pulse (1000 G for ∼10 ms) and rotated along the Y–Z plane with a weak sinusoidal magnetic field in the Z-direction [50 G (15 Pa) at 0.3 Hz] [Fig. 1(a)]. Because the NPCs were too small (average diameter of about 100 nm) to be visualized with conventional fluorescence microscopy, we utilized a strategy of quantifying donor lifetime and ratiometric (donor and acceptor) intensity FRET^38^ spectroscopies of individual NUPs in the NPCs as an index of displacements of NUPs in response to external stress. Published results from cryoEM^19,39^ data on *Xenopus laevis* have revealed that the NUP214–NUP88–NUP62 complex forms a defined resting architecture: a 9-nm three-stranded coiled-coil that connects the inner and outer Y complexes, and a second 5-nm coiled-coil subcomplex that contacts the β-propeller and outer NUP85. From a published report^18^ using Cryo-ET (Cryo-Electron Tomography) on human NPCs, we estimated that in a dilated NPC, the distance between NUP88 and NUP62 in NUP214 complex was ∼8 nm, and the distance between NUP88 and NUP43 or NUP88 and NUP133 was greater than 10 nm [Fig. 1(b)]. In contrast, the distance between NUP62 and NUP88 was ∼6 nm under resting (constricted) conditions.^18^ Considering these relative distances, three pairs of plasmid DNAs were designed with C-terminal fluorescent tags of mCFP for nucleoporins NUP88 and mYFP for NUP62, NUP43, and NUP133. We chose C-terminal tagging because many nucleoporins contain functionally important N-terminal domains involved in NPC targeting, membrane interaction, or assembly. Previous studies have shown that perturbing these terminal regions can alter nucleoporin localization and NPC function. Therefore, C-terminal tagging is commonly used as a less disruptive strategy for fluorescence imaging and FRET measurements.^40,41^ A co-transfection (a single transient transfection with two plasmid DNAs) strategy was used with two nucleoporin plasmids, which allowed the simultaneous transfection of two separate molecules into a single cell. Approximately 60%–70% co-transfection efficiency of 100 000 cells per dish was achieved. CHO (Chinese hamster ovary) cells were first grown on a fibronectin-coated (5 *μ*g/ml) dish for 12 h. CHO cells were then co-transfected with mCFP-NUP88 (donor) and mYFP-NUP62 (acceptor), or mCFP-NUP88 (donor) and mYFP-mNUP43 (acceptor), or mCFP-NUP88 (donor) and mYFP-NUP133 (acceptor) for 12 h [Fig. 1(c)]. CHO cells with nuclear stain Hoechst overlay images showed that the transfected nucleoporins NUP88, NUP62, NUP43, and NUP133 were located on the nuclear envelope [Fig. 1(c)]. Additional experiments further confirmed that transfected CFP-NUP88 and YFP-NUP62 were indeed on the nuclear envelope [supplementary material Figs. S1(A)–S1(D)]. This was examined from the overlay images of fluorescent wheat germ agglutinin (WGA) (a nuclear pore blocker,^42,43^ 50 *μ*g ml^−1^ for 30 min) in the digitonin (20 *μ*g/ml)-containing permeabilization buffer and Hoechst or CFP-NUP88-YFP-NUP62 [supplementary material Figs. S1(A) and S1(B)]. Additional experiments with overlay images of Hoechst-WGA-CFP NUP88 and YFP-NUP62 or Lamin A/C-CFP-NUP88 and Lamin A/C-YFP-NUP62 showed the locations of these NUPs on the nuclear envelope [supplementary material Figs. S1(C) and S1(D)]. We analyzed only those nuclei where both proteins, CFP-NUP88 and YFP-NUP62 (or CFP-NUP88 and YFP-NUP133 or CFP-NUP88 and YFP-NUP43), were visible at the same site, because at some sites, only one protein was bright enough to be visualized. RGD-peptide-coated magnetic beads were added at a low concentration (About 2 × 10^5^ beads were added for around 100 000 cells) to the cells (so that there was only one bead per cell) for 20 min to have sufficient time for focal adhesion formation, and loosely bound beads were washed out with medium [Fig. 1(c) insets]. In response to a transient application (<30 s) of cyclic sinusoidal bead stress (15 Pa at 0.3 Hz), FRET efficiency of YFP-NUP62 to CFP-NUP88 exhibited cyclic and dynamic changes [Fig. 1(d)], indicating that there were stress-induced changes in distances between NUP88 and NUP62. As control, under the same experimental conditions, donor bleed-through (donor bleed-through coefficient α = fraction of CFP emission detected in the FRET channel) and acceptor direct excitation (β = fraction of YFP fluorescence excited directly by the donor excitation wavelength and detected in the FRET channel) were quantified from cells expressing donor (CFP)-only or acceptor (YFP)-only constructs [supplementary material Figs. S1(E) and S1(F)]. The quantified donor bleed-through coefficient (α), corresponding to CFP emission detected in the FRET channel, was 0.10 ± 0.015 (mean ± s.d.m.), whereas the acceptor direct excitation coefficient (β), corresponding to direct excitation of YFP by donor excitation light, was 0.008 ± 0.0037 (mean ± s.d.m.) [supplementary material Fig. S1(G)]. These results suggest that the bleed-through was minimal and the detected FRET signals were mostly the result of energy transfers between donor and acceptor.

**FIG. 1.**
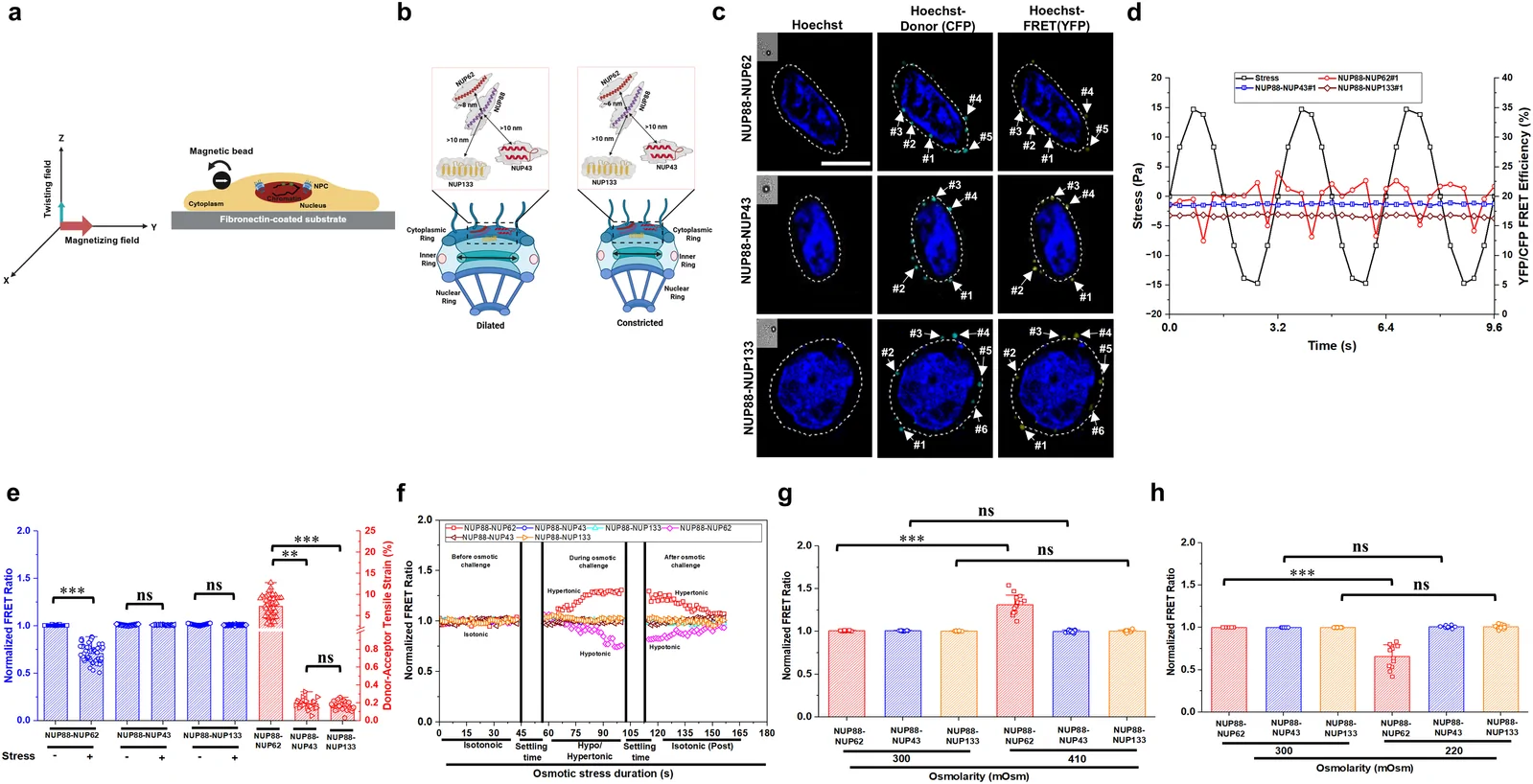
Nucleoporins exhibit dynamic deformation in response to transient local and global stresses. (a) Schematic of local stress application to NPCs of a CHO (Chinese Hamster Ovary) cell by magnetizing an RGD (arginine–glycine–aspartic acid) coated 4-*μ*m ferromagnetic bead attached to the cell apical surface in the Y-direction and twisted in the Z-direction with a sinusoidal stress (15 Pa at 0.3 Hz) using MTC (magnetic twisting cytometry). (b) Schematic of the putative relative distances among nucleoporins (NUPs) (NUP62, NUP88, NUP43, and NUP133) for dilated and constricted NPCs.^22,28,29^ (c) Representative images of brightfield (insets), Hoechst nuclear stain, overlay of Hoechst and co-transfected FRET [fluorescence resonance energy transfer; donor (CFP) and FRET (YFP)] images of CHO cells with NUP88 (monomeric CFP, mCFP; donor), NUP62 (monomeric YFP, mYFP; acceptor), NUP43 (mYFP; acceptor), and NUP133 (mYFP; acceptor) grown on rigid glass (modulus ∼2 GPa). Dotted line denotes the nuclear border, and different numbers indicate the locations of labeled nucleoporins in co-transfected cells. Scale bar, 10 *μ*m. (d) Representative original data of dynamic FRET efficiency (ratio of YFP to CFP) in response to a cyclic sinusoidal wave of applied stress (15 Pa at 0.3 Hz) for co-transfected NUPs NUP88–NUP62#1, NUP88–NUP43#1, and NUP88–NUP133#1 in the same three cells as in Fig. 1(c). (e) Normalized FRET ratio with and without applied stress and donor–acceptor tensile strain [**ε = (**R** − **R_0_)/R_0_; R and R_0_ are the apparent donor–acceptor distance with and without stress]. n = 54 transfected nucleoporins from 16 cells for NUP88–NUP62 from five independent biological experiments; n = 20 transfected nucleoporins from 7 cells for NUP88–NUP43 from three independent biological experiments; and n = 29 labeled nucleoporins from 6 cells for NUP88–NUP133 from three independent biological experiments. (f) Dynamic changes in the normalized FRET ratio of CFP NUP88–YFP NUP62, CFP NUP88–YFP NUP43, and CFP NUP88–YFP NUP133 in isotonic medium (300 mOsm; 0 to 42.5 s), in hypo-osmotic medium (220 mOsm; 57 to 99 s), or in hypertonic medium (0.1 M PEG 300; 410 mOsm; 57 to 99 s). The settling time was 15 s between medium switching. (g) Normalized FRET ratio in isotonic or hypertonic medium. n = 15 labeled nucleoporins in transient co-transfection from 5 cells for NUP88–NUP62, n = 14 labeled nucleoporins for 5 cells from NUP88–NUP43, and n = 10 labeled nucleoporins from 5 cells for NUP88–NUP133 in hypertonic medium from three independent biological experiments. (h) Normalized FRET ratio in isotonic or hypotonic medium. n = 12 labeled nucleoporins from 5 cells for NUP88–NUP62, NUP88–NUP43, NUP88–NUP133 in hypotonic medium from three independent biological experiments. Mean ± s.d.m. in E, G, and H. ^***^P *<* 0.001; ns, not statistically different. All P values were quantified using One-way ANOVA with Tukey's test and the Mann–Whitney test.

Because the exact distance between nucleoporins in an intact living cell was difficult to quantify, we estimated the “apparent” donor–acceptor distance R [quantified from efficiency equation, R = R_0_( $\frac{1}{E}-$1)^1/6^; E is the quantified FRET efficiency, and R_0_ is the Förster distance (4.9 nm for CFP-YFP)^44,45^]. As such, we used changes in FRET efficiency in response to local bead stress to estimate changes in the apparent distance between NUP88 and NUP62 [supplementary material Figs. S2(A) and S2(B)]. In contrast, there were no detectable changes in FRET efficiency and in the apparent distance between CFP-NUP88 and YFP-NUP43 or between CFP-NUP88 and YFP-NUP133 [Fig. 1(d); supplementary material Figs. S2(A), S2(C), and S2(D)], suggesting that the actual distances between these protein pairs were too large to be used for the FRET approach; hence, these two protein pairs were used as negative controls for FRET. These results were consistent with the published CryoEM data on the estimated distances between NUP88 and NUP43 or NUP133.^18,19,39^ When the FRET efficiency of YFP-NUP62 to CFP-NUP88 decreased, it indicated that the distance between the donor–acceptor (protein pairs) became farther away from each other. This decrease in FRET was taken as an index of nucleoporin stretching. Interestingly, during each cycle of the sinusoidal stress, two FRET peaks were lower than the baseline control for the NUP88–NUP62 pair [Fig. 1(d)], demonstrating that this protein pair was stretched twice when the bead was rotating back and forth from the zero-stress position. This suggests that the local surface rotational stress was transmitted onto the nuclear envelope to deform the nucleoporins. Due to the viscoelastic nature of the cytoskeleton, there was a phase lag between the input—external stress and the output—deformation of nucleoporins NUP88 and NUP62 [Fig. 1(d)]. Averaging the maximum reduction in FRET efficiency over 5 stress cycles, we measured changes in the relative FRET efficiency and the calculated strain. The tensile strain [ε = (R − R_0_)/R_0_; R and R_0_ are the apparent donor–acceptor distances with and without stress] was 7.2%, calculated from the 30% reduction in normalized FRET ratios of CFP NUP88–YFP NUP43 pair [Fig. 1(e)] in response to 16-s local bead stress. In contrast, there were no changes in normalized FRET ratios and extremely low tensile strains between NUP88 and NUP43 or NUP88 and NUP133, confirming that these protein pairs were too far away from each other for FRET [Fig. 1(e)].

Next, we exposed cells to transient global osmotic stress by switching to a hypotonic medium (addition of 30% deionized water (DI) water; 220 mOsm) or a hypertonic medium (addition of 0.1 M PEG; 410 mOsm) for the cells.^35,46^ In comparison to the isotonic condition, where there was no change in the FRET ratio in the cell [Fig. 1(f) left panel], a transient application (<45 s) of hypotonic osmotic stress resulted in a gradual reduction in the FRET ratio for NUP88–NUP62; a transient application of hypertonic osmotic stress, on the other hand, led to a gradual elevation in the FRET ratio for NUP88–NUP62 [Fig. 1(f) middle panel], suggesting that this NUP protein pair was stretched in hypotonic stress and compressed in hypertonic stress. Returning the cells to the isotonic medium resumed the FRET ratio to the prior osmotic challenge levels without memory [Fig. 1(f) right panel]. The summarized data during the osmotic challenges at 42 s show that the FRET ratios of NUP88–NUP62 increased by 31% in hypertonic stress [Fig. 1(g)] and decreased by 34% in hypotonic stress [Fig. 1(h)]; as expected, no changes in the FRET ratio were observed for NUP88–NUP43 or NUP88–NUP133 protein pairs [Figs. 1(g) and 1(h)]. Changes in apparent donor–acceptor distances between NUP88 and NUP62 were observed in response to hypertonic or hypotonic stress [supplementary material Figs. S2(E) and S2(F)]. In contrast, there were no distance changes between NUP88 and NUP43 or NUP88 and NUP133 [supplementary material Figs. S2(E) and S2(F)]. Hypertonic stress and hypotonic stress caused 6% compressive strain and 10% tensile strain, respectively, for NUP88 and NUP62 [supplementary material Figs. S2(G) and S2(H)]. However, almost no strains were detectable between the protein pair of NUP88 and NUP43 or of NUP88 and NUP133 [supplementary material Figs. S2(G) and S2(H)]. These results show that transient application of local stresses via integrins or global stresses via osmotic challenges induces transient dynamic changes in nucleoporin FRET, distances, and strains, suggesting NPCs exhibit transient dynamic changes in their sizes in response to the external stress.

### Nucleoporins exhibit mechanomemory after a short duration of global and local stress

To determine if there was mechanomemory after cessation of applied osmotic stress, we used a 5-min duration after osmotic challenges as a time window for mechanomemory because 5-min was much longer than the viscoelastic time constant (∼1 to 10 s)^47^ of the nuclear envelope. Then we asked what the minimum duration of stress application was to induce the memory effect on the nucleoporins. When the applied osmotic stress was 42 s or shorter, the NUP88–NUP62 pair did not exhibit memory of the FRET ratio change because FRET ratios 5 min post-hyperosmotic (410 mOsm) or post-hypoosmotic stress (220 mOsm) were similar to those before osmotic challenges [supplementary material Figs. S3(A) and S3(B)]. In contrast, the normalized FRET ratio remained lower for hypo-osmotic or higher for hyper-osmotic conditions for 30 min than the baseline control when the duration of osmotic stress was 60 s [supplementary material Figs. S3(C) and S3(D)], suggesting that 60-s osmotic stress duration was the minimum duration to elicit the nucleoporin memory effect. Next, we asked whether NUPs exhibit memory of deformation after a 30-min osmotic challenge to make sure that osmotic stress duration was much longer than the 60-s threshold. The normalized FRET ratio of YFP NUP62 to CFP NUP88 increased by ∼58% immediately after a 30-min hyperosmotic stress, suggesting that the separation distance between donor–acceptor NUPs decreased (an index of NUP compression); it remained ∼31% higher than the baseline level 30 min post-hyperosmotic stress and returned to the baseline level after 60 min post-hyperosmotic stress [Figs. 2(a) and 2(b)]. Compressive strain quantified from distance changes associated with the FRET efficiency showed that hypertonic stress induced NUP deformation memory, even 30 min after stress cessation [Fig. 2(c)]. In contrast, the normalized FRET ratio decreased by 32% right after a 30-min hypoosmotic stress and remained 20% lower at 30 min post-hypoosmotic stress, suggesting that the separation distance between donor–acceptor NUPs increased (an index of NUP stretching) and remained elevated; the FRET levels returned to the baseline after 60 min post-hypoosmotic stress [Figs. 2(d) and 2(e)]. Further estimation of tensile strain from distance changes associated with this FRET efficiency confirmed NUP deformation memory induced by the hypotonic stress [Fig. 2(f)]. There was no change in normalized FRET ratio in the 60-min temporal control in isotonic cell culture medium (∼300 mOsm) [supplementary material Fig. S3(E)]. These results suggest that the NUP88–NUP62 protein pair exhibits deformation mechanomemory after cessation of osmotic stress for 60 s or longer (30 min).

**FIG. 2.**
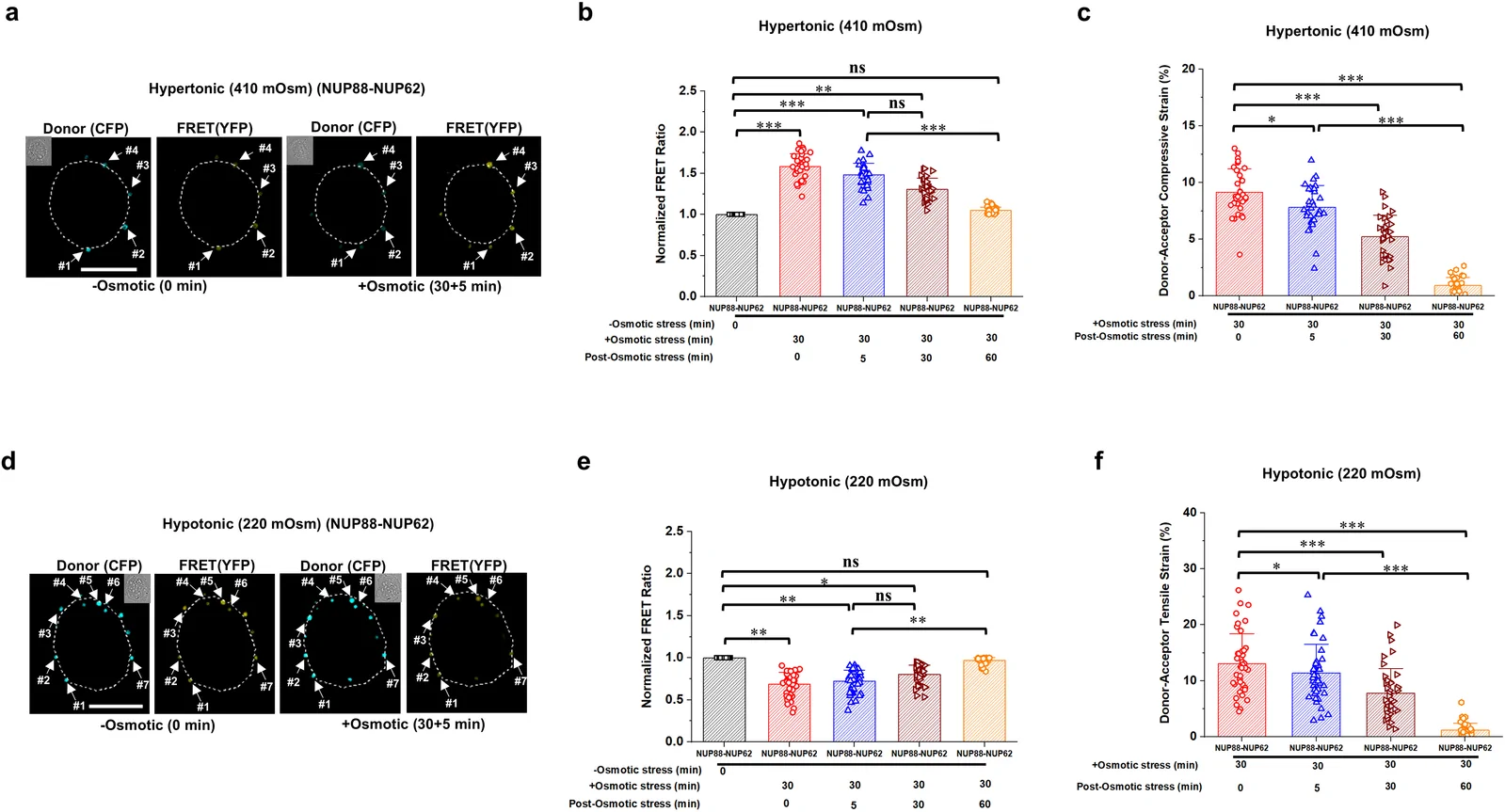
Nucleoporins exhibit mechanomemory after a short duration of global osmotic stress. (a) Representative brightfield (small insets) and fluorescent images of CFP NUP88 (donor) and YFP NUP62 (acceptor) of CHO cells grown on rigid glass (2 GPa) in isotonic (−Osmotic stress; cell culture medium; ∼300 mOsm), and 5 min after 30 min hypertonic (+Osmotic stress; addition of 0.1 M PEG in medium; ∼410 mOsm) osmotic stress (post-isotonic; after washing hypertonic and adding isotonic cell culture medium). Dotted line denotes the nuclear border, and different numbers indicate the locations of labeled nucleoporins in co-transfected cells. Scale bar, 10 *μ*m. (b) Normalized FRET ratio of YFP NUP62 to CFP NUP88 in isotonic medium, 30-min hypertonic medium, switching back to isotonic medium for 0, 5, 30, or 60 min after washing out hypertonic medium. n = 32 labeled nucleoporins after transient co-transfection from 7 cells for NUP88–NUP62 from three independent experiments. (c) Donor–acceptor compressive strain after being exposed to a 30-min hypertonic medium and then switching to an isotonic medium for 0, 5, 30, or 60 min. n = 32 labeled nucleoporins from 7 cells for NUP88–NUP62 from three independent experiments. (d) Representative brightfield (small insets) and fluorescent images of CFP NUP88 (donor) and YFP NUP62 (acceptor) of CHO cells grown on rigid glass (2 GPa) in isotonic (−Osmotic stress; cell culture medium; ∼300 mOsm), and 5 min after hypotonic (+Osmotic stress; addition of 30% DI water in medium; ∼220 mOsm), osmotic stress (post-isotonic; after washing hypotonic and adding isotonic cell culture medium). Dotted line denotes the nuclear border, and different numbers indicate the locations of labeled nucleoporins in co-transfected cells. Scale bar, 10 *μ*m. (e) Normalized FRET ratio of YFP NUP62 to CFP NUP88 in isotonic medium, hypotonic medium at 30 min, post-isotonic medium at 5, 30, and 60 min after washing out hypotonic medium. n = 41 labeled nucleoporins, from 11 cells for NUP88–NUP62 from three independent experiments. (f) Donor–acceptor tensile strain after exposure to a 30-min hypotonic medium, then switching back to an isotonic medium for 0, 5, 30, or 60 min. n = 41 labeled nucleoporins from 7 cells for NUP88–NUP62 from three independent experiments. ^*^P < 0.05, ^**^P < 0.01, ^***^P < 0.001; ns, not statistically different. All P values were quantified using One-way ANOVA with Tukey's test and the Mann–Whitney test.

Because 60 s was also the minimum duration of external bead stress to induce stretching mechanomemory [supplementary material Figs. S3(F)–S3(H)], we chose to apply a local magnetic bead stress via integrins for 10 min and determined its effect on nucleoporins. NUP88–NUP62 exhibited stretching mechanomemory for 30 min after cessation of a 10-min bead stress (15 Pa at 0.3 Hz) when the cells were plated on FN-coated rigid glass [Figs. 3(a)–3(d)]. Using the approach of FLIM (fluorescence lifetime imaging microscopy**)**-FRET imaging, we found that the FRET efficiency of NUP88 (donor) and NUP62 (acceptor) decreased from ∼20% prior stress to ∼8% 5 min after cessation of the 10-min bead stress, a 60% reduction in FRET efficiency; the FRET efficiency returned to the baseline value by 60 min post stress [supplementary material Fig. S4(A)]. The donor–acceptor tensile strain remained elevated at ∼18.7% 5 min after stress cessation and was reduced to ∼2.9% 60 min post-stress [supplementary material Fig. S4(B)]. As a control for fluorescent lifetime FLIM-FRET imaging, the NUP88 donor only did not exhibit memory after cessation of the applied bead stress [supplementary material Fig. S4(C)], suggesting that there was stretching mechanomemory in the NUP88–NUP62 protein pair. These results are consistent with the fluorescence intensity FRET data in Figs. 3(a)–3(d). Comparing 5 min post 10-min stress data from Fig. 3(d) and supplementary material Fig. S4(B), we find that the tensile strains were similar from intensity FRET and from lifetime FRET, reinforcing the observation that stress induces stretching mechanomemory in NUPs in the NPC. Together, the data suggests that nucleoporins exhibit deformation mechanomemory after short durations of global osmotic stress (for 30 min) and of local bead stress (for 10 min).

**FIG. 3.**
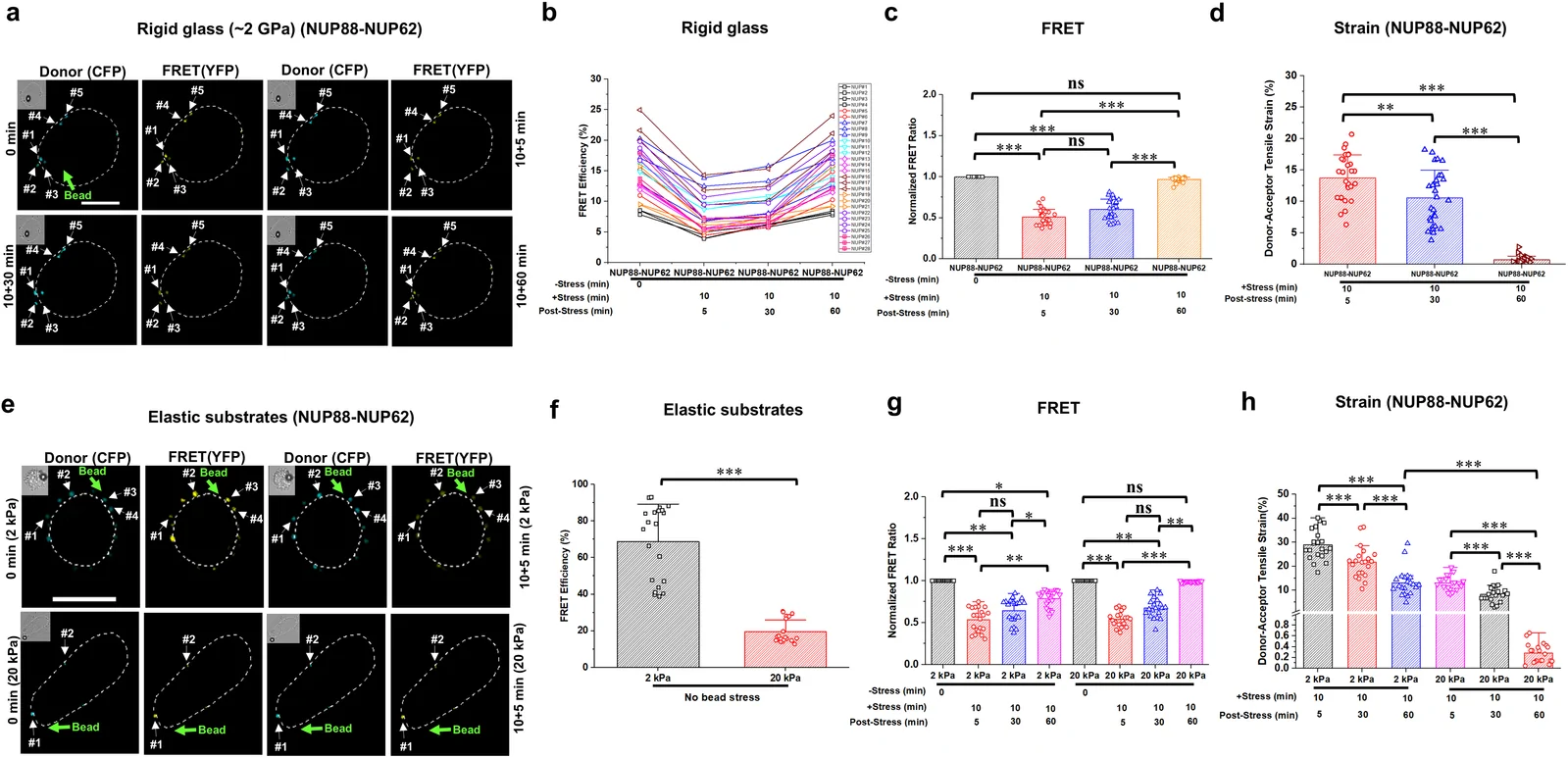
Mechanomemory of nucleoporin stretching after cessation of applied local stress to cells on rigid and elastic substrates. (a) Representative brightfield (small insets) and Lipofectamine-based co-transfected FRET images of CFP NUP88 (donor) and YFP NUP62 (acceptor) of CHO cells grown on rigid glass (2 GPa) at no stress control, 5, 30, and 60 min after 10-min stress (15 Pa at 0.3 Hz). Dotted line denotes the nuclear border, and different numbers indicate the locations of labeled nucleoporins in co-transfected cells. Scale bar, 10 *μ*m. (b) FRET efficiency of YFP NUP62 to CFP NUP88 at no stress, 5, 30, and 60 min after 10-min stress. Data are shown as a line graph where each transfected NUP was tracked in all four conditions. n = 28 labeled nucleoporins from 9 cells for NUP88–NUP62 from four independent biological experiments. (c) Normalized FRET ratio of YFP NUP62 to CFP NUP88. n = 28 labeled nucleoporins from 9 cells for NUP88–NUP62 from four independent biological experiments. (d) Donor–acceptor tensile strain between no stress and 5, 30, or 60 min after stress cessation. n = 28 labeled nucleoporins from 9 cells for NUP88–NUP62 from four independent biological experiments. (e) Representative brightfield (BF), and Lipofectamine-based co-transfected FRET images of CFP NUP88 (donor) and YFP NUP62 (acceptor) of CHO cells grown on polyacrylamide (PA) elastic substrates of 2 and 20 kPa at no stress control and 5 min after 10-min stress. Dotted line denotes the nuclear border, and different numbers indicate the locations of labeled nucleoporins in co-transfected cells. Scale bar, 10 *μ*m. (f) FRET efficiency of YFP NUP62 to CFP NUP88 in co-transfected CHO cells grown on elastic 2 and 20 kPa PA gel at no stress. n = 21 labeled nucleoporins from 5 cells for NUP88–NUP62 for 2 kPa and n = 20 labeled nucleoporins from 5 cells for NUP88–NUP62 for 20 kPa from three independent biological experiments. (g) Normalized FRET ratio of YFP NUP62 to CFP NUP88 in CHO cells at various times after 10 min stress for cells grown on elastic 2 and 20 kPa gel. n = 21 labeled nucleoporins from 5 cells for 2 kPa and n = 20 labeled nucleoporins from 5 cells for 20 kPa from three independent biological experiments. (h) Donor–acceptor tensile strain between no stress and 5, 30, or 60 min post-stress for cells grown on elastic 2 and 20 kPa substrates. n = 21 labeled nucleoporins from 5 cells for NUP88–NUP62 for 2 kPa and n = 20 labeled nucleoporins from 5 cells for NUP88–NUP62 for 20 kPa from three independent biological experiments. All data are shown as a bar plot with mean ± s.d.m. in c, d, f, g, and h. ^*^P < 0.05, ^**^P < 0.01, ^***^P < 0.001; ns, not statistically different. All P values were quantified using One-way ANOVA with Tukey's test and the Mann–Whitney test.

### Soft elastic and fast-relaxing viscoelastic substrates prolong NUP mechanomemory

Next, we asked how the elasticity and viscoelasticity of the substrate might modulate the duration of external stress-induced nucleoporin mechanomemory. When the cells were plated on FN-coated elastic substrates, in the absence of external stress, the FRET efficiency of YFP-NUP62 to CFP NUP88 was higher on the soft 2-kPa substrate than on the stiff 20-kPa substrate [Figs. 3(e) and 3(f)], suggesting that the nucleoporin pair was closer to each other on the soft substrate of the 2-kPa substrate than on the 20-kPa substrate. After 10-min bead stress, the normalized FRET ratio on the 2-kPa substrate decreased by ∼47%, 5 min after stress cessation when compared with the no-stress control; the normalized FRET ratio remained 22% lower even 60 min after stress cessation [Fig. 3(g); supplementary material Fig. S4(D)]; the calculated tensile strain was ∼30% 5 min post-stress and remained at 13.2% even 60 min post-stress [Fig. 3(h)]. In contrast, tensile strain decreased on the 20-kPa substrate when compared to on the 2-kPa substrate, and the FRET ratio of NUP88–NUP62 on the 20-kPa substrate returned to the baseline level 60 min after stress cessation [Figs. 3(g) and 3(h); supplementary material Fig. S4(E)]. In the absence of applied bead stress, there was no change in normalized FRET ratio for the temporal control of 10 min on 2 or 20 kPa elastic substrates [supplementary material Figs. S4(F) and S4(G)]. These data suggest that the soft elastic substrate extends the duration of stretching mechanomemory of NUPs, in part due to lower cytoskeletal prestress on the soft elastic substrate, resulting in lower nuclear envelope tension and higher NUP strains than on the stiff elastic substrate [Fig. 3(h)].

When the cells were plated on viscoelastic substrates, in the absence of external stress, FRET efficiency was higher for the NUP88–NUP62 pair on the fast relaxing (relaxation half time of ∼200 s) 2-kPa substrate than on the slow relaxing (relaxation half time of ∼1000 s) 2-kPa substrate [Figs. 4(a) and 4(b)]; in contrast, there was no difference in FRET efficiency between the slow and the fast relaxing substrates on the 20-kPa substrate [Figs. 4(c) and 4(d)]. FRET efficiency was inversely associated with the cell spreading area on the elastic substrates: the cells spread more on the 20-kPa elastic substrate than on the 2-kPa substrates, and the FRET efficiency was lower on the 20-kPa elastic substrate [Figs. 4(e) and 4(f)]. On 2-kPa substrates, the FRET efficiency was also inversely associated with the cell spreading area on the viscoelastic substrates: the cells spread less on the fast-relaxing than on the slow-relaxing, and FRET efficiency of NUP88–NUP62 was higher on the fast-relaxing substrate [Figs. 4(e) and 4(f)]; in contrast, on the 20-kPa substrates, there was no difference in cell spreading between slow- and fast-relaxing 20-kPa substrates, and no difference in FRET efficiency was observed [Figs. 4(e) and 4(f)], suggesting that the rigidity of the substrates overrides the effect of relaxation time under these conditions. When the substrate elasticity (20 kPa) was much higher (>10-fold) than the cell stiffness (∼1 kPa), the cells would be pulled to spread on a stiff substrate, based on the molecular clutch model.^48^ As a result, the dominant factor would be the cell's viscoelasticity itself, and the relaxation time (fast or slow) of the substrate would play a minor role. This interpretation is consistent with results that the same cell spreading area is associated with the same FRET ratio [Fig. 4(f)].

**FIG. 4.**
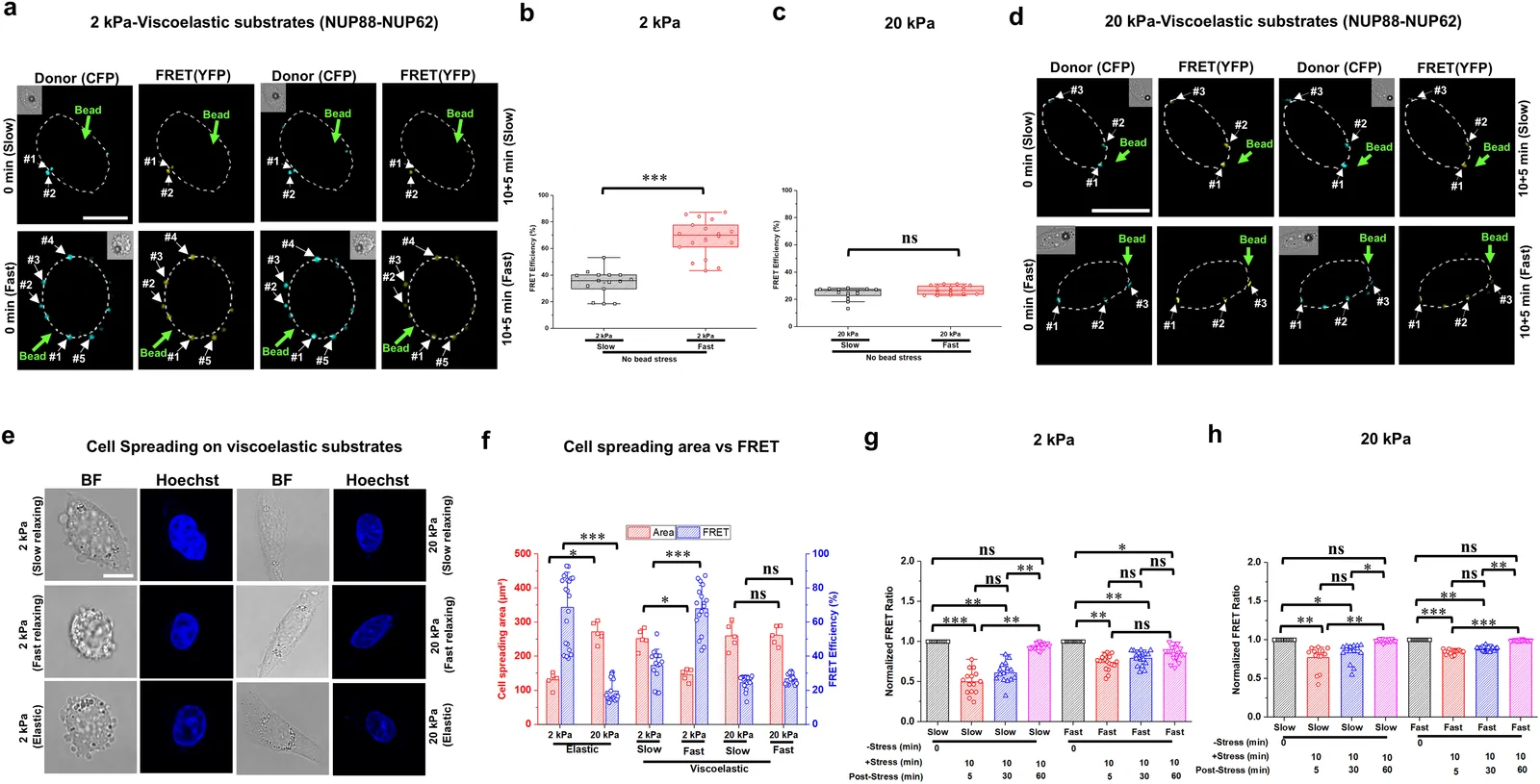
Soft fast-relaxing viscoelastic substrates prolong duration of stress-induced NUP mechanomemory, and FRET efficiency is inversely associated with cell spreading areas on elastic and viscoelastic substrates. (a) Representative brightfield (BF) and Lipofectamine-based co-transfected FRET images of CFP NUP88 and YFP NUP62 in CHO cells grown on slow (relaxation half times ∼1000 s) and fast (relaxation half times ∼200 s) relaxing alginate viscoelastic substrates of 2 kPa at no stress control and 5 min after 10-min stress (15 Pa at 0.3 Hz). Dotted line denotes the nuclear border, and different numbers indicate the locations of labeled nucleoporins in co-transfected cells. Scale bar, 10 *μ*m. (b) FRET efficiency of YFP NUP62 to CFP NUP88 on 2 kPa slow and fast relaxing viscoelastic substrates without stress. Data are shown as a boxplot with values of minimum, 5% percentile, 25% percentile, median, 75% percentile, maximum, and mean. n = 15 labeled nucleoporins NUP88–NUP62 from 5 cells for slow and n = 18 labeled nucleoporins NUP88–NUP62 from 5 cells for fast relaxing 2 kPa substrates from three independent biological experiments. (c) FRET efficiency of CHO cells grown on viscoelastic 20 kPa slow and fast relaxing substrates without stress; n = 15 labeled nucleoporins NUP88–NUP62 from 5 cells for slow and n = 18 labeled nucleoporins NUP88–NUP62 from 5 cells for fast relaxing 20 kPa substrates from three independent biological experiments. (d) Representative brightfield (BF) and FRET images of CFP NUP88 and YFP NUP62 of CHO cells grown on slow and fast relaxing alginate viscoelastic substrates of 20 kPa at no stress and 5 min after stress. Dotted line denotes the nuclear border, and different numbering indicates the locations of labeled nucleoporins in co-transfected cells. Scale bar, 10 *μ*m. (e) Representative brightfield (BF) and nuclear stain (Hoechst) images of CHO cells grown on elastic, slow, and fast relaxing viscoelastic substrates of 2 and 20 kPa. Scale bar, 10 *μ*m. (f) FRET efficiency of YFP-NUP62 to CFP-NUP88 and spreading area of CHO cells grown on various elastic or viscoelastic substrates. Mean ± s.d.m.; n = 21 labeled nucleoporins NUP88–NUP62 from 5 cells on 2-kPa elastic; n = 20 labeled nucleoporins NUP88–NUP62 from 5 cells on 20-kPa elastic; n = 15 labeled nucleoporins NUP88–NUP62 from 5 cells on 2-kPa slow viscoelastic; n = 18 labeled nucleoporins NUP88–NUP62 from 5 cells on 2-kPa fast viscoelastic; n = 15 labeled nucleoporins NUP88–NUP62 from 5 cells on 20-kPa slow viscoelastic; n = 18 labeled nucleoporins NUP88–NUP62 from 5 cells on 20-kPa fast viscoelastic; thee independent biological experiments. (g) Normalized FRET ratio of YFP NUP62 to CFP NUP88 in CHO cells at no stress or 5, 30, and 60 min after stress for cells grown on slow and fast relaxing viscoelastic 2 kPa substrates. n = 15 labeled nucleoporins NUP88–NUP62 from 5 cells for slow and n = 18 labeled nucleoporins NUP88–NUP62 from 5 cells for fast relaxing 2 kPa for YFP NUP62-CFP NUP88 from three independent biological experiments. (h) Normalized FRET ratio for cells grown on slow- and fast-relaxing viscoelastic 20 kPa substrates. n = 15 labeled nucleoporins NUP88–NUP62 from 5 cells for slow-relaxing and n = 18 labeled nucleoporins NUP88–NUP62 from 5 cells for fast-relaxing, from three independent biological experiments. ^*^P < 0.05, ^**^P < 0.01, ^***^P < 0.001; ns, not statistically different. All P values were quantified using One-way ANOVA with Tukey's test and the Mann–Whitney test.

After application of a 10-min bead stress, the normalized FRET ratio reduction was more pronounced on the 2 kPa slow-relaxing viscoelastic substrate (50% at 5 min post-stress) than on the fast-relaxing substrate (21% at 5 min post-stress) [Fig. 4(g); supplementary material Fig. S4(H)]. However, on soft 2-kPa substrates, in comparison to the NUPs on the slow-relaxing substrate, the mechanomemory of NUPs stretching on the fast-relaxing substrate remained longer than 60 min after cessation of the bead stress [Fig. 4(g); supplementary material Fig. S4(H)]. In contrast, on stiff 20-kPa substrates, there was no difference in normalized FRET ratio for NUPs between slow- and fast-relaxing substrates 60 min post-stress application [Fig. 4(h); supplementary material Fig. S4(I)]. In the absence of stress, there was no change in normalized FRET ratio for the temporal time control of 10 min on both slow- and fast-relaxing viscoelastic substrates [supplementary material Figs. S4(J) and S4(K)]. These results suggest that greater distance separated on soft (2 kPa), and viscous (fast-relaxing) substrates explains the slower return to the baseline, facilitating longer durations of stretching-induced mechanomemory of nucleoporins in the NPCs.

### Mechanomemory of NPC sizes quantified by MINFLUX after stress cessation

The results from FRET experiments in nucleoporin deformation mechanomemory suggest that there should be mechanomemory of the NPC size after mechanical stimulation because nucleoporins are the building blocks of the NPC. To directly visualize and quantify changes of NPC size in a living cell plated on a rigid glass, we employed one-color MINFLUX nanoscopy technology.^23^ U2OS-CRISPR-NUP96 cells were labeled with haloTag SaraFluor 650B ligand^49^ [supplementary material Fig. S5(A)]. We used the MINFLUX technique because it could resolve 1–3 nm spatial resolution.^22^ Here, halo-tagged U2OS NUP96 cells were seeded on a glass-bottom dish and incubated for 1 h with SaraFluor 650B ligand at 37 °C and 5% CO_2_. After that, cells were washed three times, each for 1 h, to remove free dyes deposited on the dish and reduce the background noise. During MINFLUX imaging, background noise was distinguished from stained NUP96 by setting the lower threshold value of effective frequency at offset (EFO) value to 25 kHz and the upper limit of EFO to 80 kHz [supplementary material Fig. S5(B)]. This lower limit of EFO values distinguishes NPC localization data of the SaraFluor 650B ligand from the background noise. A CFR (Center-Frequency Ratio) value of 0.8 was used to filter the raw MINFLUX data [supplementary material Fig. S5(C)]. An Abberior instrument of the MINFLUX microscope with 642-nm excitation (continuous wave) activation lasers was used for imaging of NPCs. To minimize drift during measurements, 150-nm positively charged gold nanoparticles [lower left corner of the top left panel in Fig. 5(a)] were deposited on a 35-mm glass-bottomed dish and used as a reference on the bottom of the cells. For all experiments, drift was minimized with the MINFLUX built-in stabilization system and maintained at <0.8 nm in XYZ. All MINFLUX imaging was performed at the basal nuclear envelope of the cell nucleus and stabilized by using the gold nanoparticle.

**FIG. 5.**
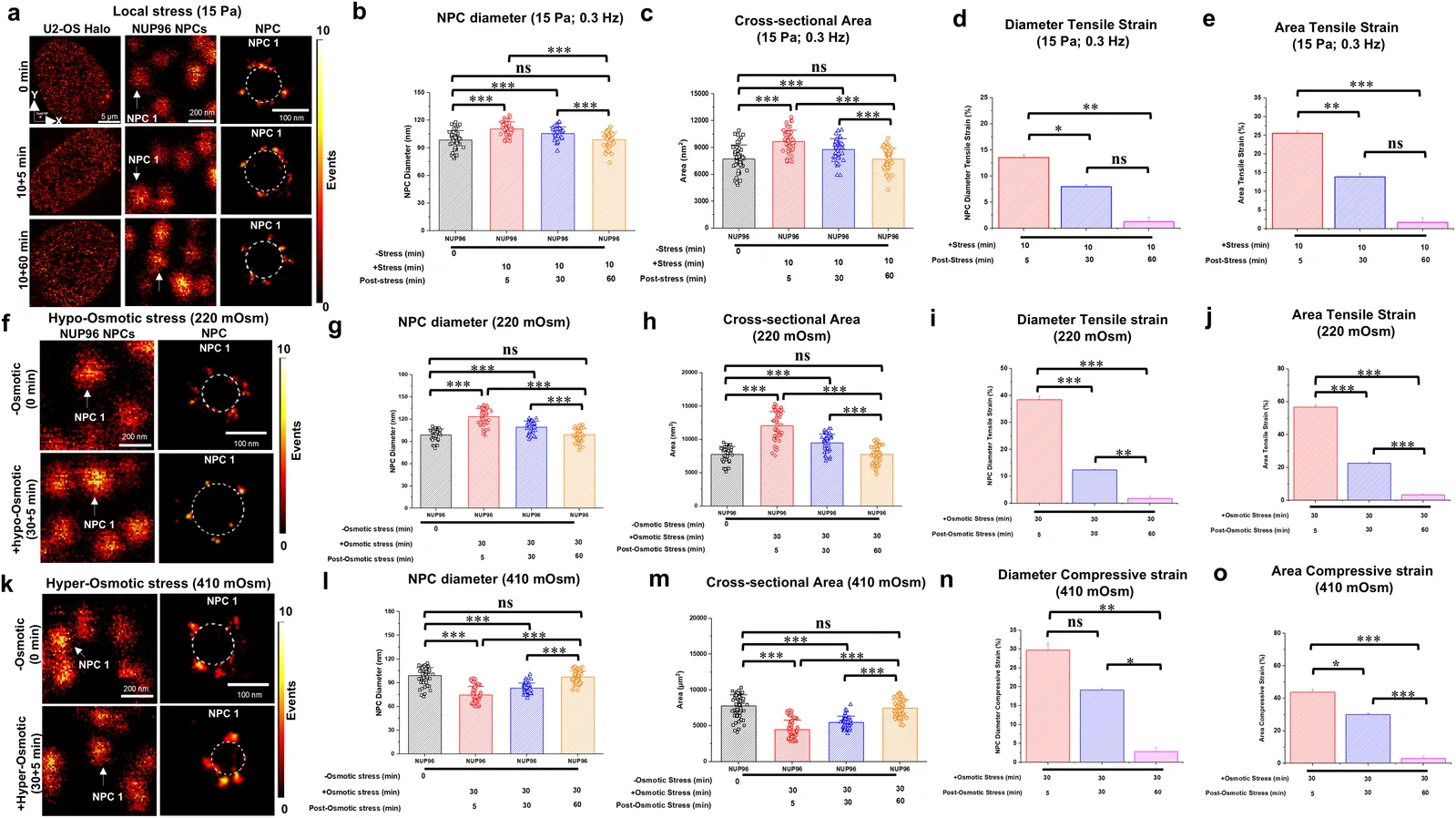
Mechanomemory of NPC sizes after cessation of local and global stress. (a) Representative MINFLUX images of live U2OS-CRISPR-NUP96 Halo cells labeled with halo-Tag SaraFluor 650B ligand at no stress, 5, and 60 min after stress. MINFLUX images were stabilized by using 150 nm positively charged gold nanoparticles (lower left corner of top panel). Dotted lines denote the circular fitting of MINFLUX localization data. MINFLUX image scale bars, 5 *μ*m, 200, and 100 nm, respectively. (b) Nominal diameter (diameter of a circle fitted with MINFLUX localization data) in live U2OS-CRISPR-NUP96 Halo cells. NPCs quantified from MINFLUX images at 0-, 5-, 30-, or 60-min after 10-min stress applied via RGD-coated magnetic bead on the cell apical surface. (c) Quantified cross-sectional area A = (π*(diameter/2)2) of circularly fitted nuclear pores quantified in MINFLUX at no stress (0 min), 5-, 30-, or 60-min after the application of stress of 15 Pa at 0.3 Hz. (d) Mean NPC diameter changes (tensile strain) in MINFLUX between no stress and 5-, 30-, or 60-min after the application of stress of 15 Pa at 0.3 Hz. Data presents as bar plot with mean + s.e.m. (13.57 ± 0.47, 7.95 ± 0.3, and 1.23 ± 0.87 respectively for 10 + 5, 10 + 30, and 10 + 60 min stress) for 5-, 30-, or 60- min after stress for the data as shown in (b). (e) Mean NPC cross-sectional area changes (tensile strain) in MINFLUX between no stress and 5-, 30-, or 60-min after the application of stress of 15 Pa at 0.3 Hz. Data presents as bar plot with mean + s.e.m. (25.52 ± 0.62, 13.85 ± 0.97, and 1.64 ± 1.27, respectively, for 10 + 5, 10 + 30, and 10 + 60 min stress) for 5-, 30-, or 60-min after stress for the data as shown in (c). (f) Representative MINFLUX images of intact live U2OS cells in isotonic (−Osmotic stress) and 5 min after 30 min hypo-osmotic pressure of 220 mOsm. Dotted lines denote the circular fitting of MINFLUX localization data. MINFLUX image scale bars, 200, and 100 nm, respectively. (g) Nominal diameter of live U2OS cell NPCs quantified from MINFLUX images in isotonic (300 mOsm), and 5-, 30-, or 60-min after switching to isotonic medium, after the cells were treated with hypotonic challenge (30% DI water; 220 mOsm) for 30 min. (h) Quantified cross-sectional area of circularly fitted nuclear pores quantified in MINFLUX in isotonic (300 mOsm), and 5-, 30-, or 60-min after switching to isotonic medium, after the cells were treated with hypotonic challenge (30% DI water; 220 mOsm) for 30 min. (i) Mean NPC diameter changes (tensile strain) in MINFLUX between isotonic (300 mOsm), and 5-, 30-, or 60-min after switching to isotonic medium, after the cells were treated with hypotonic challenge (30% DI water; 220 mOsm) for 30 min. Data presents as bar plot with mean + s.e.m. (38.36 ± 1.27, 12.34 ± 0.21, and 1.77 ± 0.0.93, respectively for 30 + 5, 30 + 30, and 30 + 60 min stress) for 5-, 30-, or 60-min post-hypo-osmotic stress for the data as shown in (g). (j) Mean NPC cross-sectional area changes (tensile strain) in MINFLUX between isotonic (300 mOsm), and 5-, 30-, or 60-min after switching to isotonic medium, after the cells were treated with hypotonic challenge (30% DI water; 220 mOsm) for 30 min. Data presents as bar plot with mean + s.e.m. (56.72 ± 1.58, 22.59 ± 0.54, and 3.18 ± 0.76 respectively for 30 + 5, 30 + 30, and 30 + 60 min stress) for 5-, 30-, or 60-min post-hypo-osmotic stress for the data as shown in (h). (k) Representative MINFLUX images of intact live U2OS cells at 0 min in isotonic (−Osmotic stress) and 5 min after 30 min hypertonic pressure of 410 mOsm. Dotted lines denote the circular fitting of MINFLUX localization data. MINFLUX image scale bars, 200, and 100 nm, respectively. (l) Nominal diameter of live U2OS cell NPCs quantified from MINFLUX images in isotonic (300 mOsm), and 5-, 30-, or 60-min in isotonic medium for cells treated with hypertonic (0.1 M PEG 300; 410 mOsm) medium. (m) Quantified cross-sectional area of circularly fitted nuclear pores quantified in MINFLUX in isotonic (300 mOsm), and 5-, 30-, or 60-min in isotonic medium for cells treated with hypertonic (0.1 M PEG 300; 410 mOsm) medium. (n) Diameter compressive strain in MINFLUX between isotonic (300 mOsm), and 5-, 30-, or 60-min in isotonic medium for cells treated with hypertonic (0.1 M PEG 300; 410 mOsm) medium. Data presents as bar plot with mean + s.e.m. (29.72 ± 1.83, 19.18 ± 0.37, and 2.82 ± 1.05, respectively, for 30 + 5, 30 + 30, and 30 + 60 min stress) for 5-, 30-, or 60-min post hyperosmotic stress for the data as shown in (l). (o) Area compressive strain in MINFLUX between isotonic (300 mOsm), and 5-, 30-, or 60-min in isotonic medium for cells treated with hypertonic (0.1 M PEG 300; 410 mOsm) medium. Data present as bar plot with mean + s.e.m. (43.72 ± 1.76, 30.01 ± 0.67, and 3.10 ± 1.34, respectively for 30 + 5, 30 + 30, and 30 + 60 min stress) for 5-, 30-, or 60-min post hyperosmotic stress for the data as shown in (m). n = 52 NPCs in 19 cells, n = 48 NPCs in 12 cells, n = 40 NPCs in 11 cells, and n = 48 NPCs in 11 cells at 0 min, and 5-, 30-, and 60-min after 10-min stress from five independent experiments in (b)–(e). n = 44 NPCs from 13 cells, n = 61 NPCs from 13 cells (5 min post switching), n = 47 NPCs from 10 cells (30 min post switching back to isotonic medium), and 63 NPCs from 13 cells (60 min post switching) from five independent experiments in (g)–(j). n = 64 NPCs for 12 cells in isotonic medium (0 min), n = 62 NPCs for 13 cells at 5 min after 30 min hypertonic challenge, n = 46 NPCs for 9 cells at 30 min after 30 min hypertonic challenge, and n = 60 NPCs for 13 cells at 60 min after washing hypertonic medium and adding post-isotonic medium, from five independent experiments in (l)–(o). For mean + s.e.m. in subfigures (d), (e), (i), (j), (n), and (o), n = 5 (independent experiments); each strain value is calculated from mean diameter differences at various time points post stress, divided by the mean diameter at zero stress from many NPCs from multiple cells. After that, mean strain was calculated from five different strain values from five independent experiments. ^*^P < 0.05, ^**^P < 0.01, ^***^P < 0.001; ns, not statistically different. All P values were quantified using One-way ANOVA with Tukey's test and the Mann–Whitney test.

NUP96s are located at both the cytoplasmic and nucleoplasmic rings, and each ring consists of 8 protein pairs (32 NUP96s in total per NPC). In MINFLUX, NUP96s at the cytoplasmic ring were identified and visualized to measure individual NPC diameters by using the gold nanoparticle on the dish as a reference for Z-distance. Due to SaraFluor 650B's self-blinking nature, cell dishes were first scanned via low-resolution confocal microscopy to locate potential regions of interest (ROI) [left panel in Fig. 5(a)]. A small ROI with a few NPCs was determined by scanning in confocal mode at highresolution [middle panel in Fig. 5(a)]. From this ROI, one NPC was selected and imaged in a live U2OS cell with MINFLUX, and localization data were collected with a 642-nm excitation laser [right panel in Fig. 5(a)]. These MINFLUX localization data (∼75 locations per NPC) were fitted with a circle to identify the diameter of the NPC [dashed line in right panel in Fig. 5(a)].^22^ A large set of NPC localization data from multiple intact live cells was analyzed to mitigate any stochastic variability of the localization data.

In the absence of external bead stress, the NUP96 location data were collected and fitted with a circle to quantify the NPC diameter in 2D. The average NPC diameter without stress (0 min) was determined to be 98.6 ± 10.3 nm (mean ± s.d.m.) (ranging from 78 to 118 nm) [Fig. 5(b)], similar to that (∼102 nm) of NPCs in permeabilized U2OS cells in a published report.^23^ These data are also consistent with the published data, where NPC diameters range from 90 to 135 nm in wild-type mouse embryonic stem cells.^20^ Due to the nature of the self-blinking dye, multiple different dishes were used in MINFLUX imaging of living intact cells at 5-, 30-, or 60-min after stress cessation. Five minutes after cessation of 10-min local bead stress [Fig. 5(a), middle row panel], the NPC diameter was found to be 110.7 ± 7.5 nm (ranging from 97.2 to 125.6 nm) [Fig. 5(b)], suggesting that there was stress-induced mechanomemory of the NPC dilation. MINFLUX experiments with live cells show that the average NPC diameter remained dilated at 105.5 ± 7.4 nm, 30 min after bead stress cessation [Fig. 5(b)]. However, average NPC diameter returned to the prior-stress value 60 min after stress cessation [Fig. 5(a) bottom row panel; Fig. 5(b)]. Data from a large region of 5–9 NPCs further demonstrated that stress-induced mechanomemory existed in NPC size dilation in intact living cells [supplementary material Figs. S5(D)–S5(G)].

To determine if the NPC area dilated, we quantified the cross-sectional area [A = π*(diameter/2)^2^] from the quantified diameter of the fitted circle from 2D NPC location data [Fig. 5(c)]. The cross-sectional area increased from 7712 nm^2^ before stress (0 min) to 9659 nm^2^ 5 min post 10-min stress [Fig. 5(c)]; the cross-sectional area stayed dilated even 30 min post 10-min stress, where the average area was 8788 nm^2^ [Fig. 5(c)]. The NPC area returned to the baseline level at 60 min post 10-min stress [Fig. 5(c)].

Furthermore, circumferential tensile strain [ε = (2πr − 2πr_0_)/2πr_0_; r and r_0_ are the average NPC diameter with and without stress] was quantified by averaging NPC diameter changes in MINFLUX between no stress (0 min; control) and at 5-, 30-, or 60-min after the application of local stress of 15-Pa at 0.3 Hz [Fig. 5(d)]. The results show that tensile strain was 13.6% 5 min after stress, decreased to 7.9% at 30 min after stress, and was 1.23% 60 min after stress [Fig. 5(d)], similar to the tensile strains of nucleoporins from FRET experiments in response to the local stress of the same amplitude, frequency, and duration [compare with those in Fig. 3(d)]. The NPC cross-sectional area strains increased by 25.5% and 13.9% respectively at 5- and 30-min post stress, the area strain returned to the baseline level 60 min post-stress [Fig. 5(e)]. These results show that NPCs are dilated by local stress on the cell surface via integrins.

After a global stress applied via switching to the hypo-osmotic medium of ∼220 mOsm for 30 min [Fig. 5(f)], the NPC diameter dilated [Fig. 5(g)] from 98.9 ± 8 nm in the control (isotonic) medium to 123.3 ± 11.3 nm at 5 min after switching back to the isotonic medium. The NPC diameter remained dilated at 109.4 ± 8.0 nm 30 min post-switching back to the isotonic medium. The NPC diameter eventually returned to the pre-osmotic challenge level at 60 min post-switching back [Fig. 5(g)]. The cross-sectional area increased from 7729 nm^2^ at isotonic to 12035 nm^2^ at 5 min after the hypo-osmotic stress [Fig. 5(h)]. The area dilation remained dilated 30 min after 30-min hypo-osmotic stress, as the average cross-sectional area was 9454 nm^2^ [Fig. 5(h)]. The NPC area returned to the baseline level 60 min after hypo-osmotic stress [Fig. 5(h)]. Calculated diameter tensile strain and area tensile strain were 38.4% and 56.7%, respectively, 5 min post hypo-osmotic stress. They were 12.3% and 22.6%, respectively, 30 min post hypo-osmotic stress, and almost returned to the baseline level at 60 min post hypo-osmotic stress [Figs. 5(i) and 5(j)].

In contrast, after switching to the hyperosmotic medium of ∼410 mOsm for 30 min and then switching to isotonic medium, the NPC diameter constricted [Fig. 5(k)] from 98.9 ± 10.4 to 74.5 ± 10.9 nm at 5 min post stress. NPC diameters remain constricted at 83.1 ± 6.7 nm 30 min post hyperosmotic stress in isotonic medium. NPC diameter constriction returned to the baseline 60 min post-hyperosmotic stress in isotonic medium [Fig. 5(l)]. NPC diameter and cross-sectional area compressive strains were 29.7% and 43.7% at 5-min, and 19.2% and 30%, respectively, at 30-min post-hyperosmotic stress in isotonic medium; both largely returned to the baseline level 60 min after hyper-osmotic stress [Figs. 5(m)–5(o)]. Together, these data suggest that global osmotic stress dilates or constricts NPC with a memory duration of ∼30 min for cells on the rigid glass.

### Mechanomemory of nuclear importins α and β1 translocation

Because local bead stress and global hypo- or hyper-osmotic stress induce area dilation or constriction of the NPC, we wondered if the dilated or constricted area affects the nuclear translocation of importins α and β1. Importin β1 is a critical nuclear transport receptor that transports proteins into the nucleus via binding to the proteins directly or indirectly via adaptor protein importin α.^50^ We employed two different methods to test the hypothesis that dilated NPCs facilitate importin translocation through the NPC: (1) two-color MINFLUX to track and count single-molecule importins at separate locations of NPCs, and (2) immunofluorescence staining for endogenous importins to quantify the nuclear to cytoplasmic ratio.

In two-color MINFLUX imaging, NUP96s were labeled with haloTag SaraFluor 650B ligand. NPCs were stained with the same protocol as that for 2D NPC localization to quantify their diameters in the X–Y plane. For importins α and β1 labeling, we cloned fluorescent protein plasmids (pDsRed-importin α or pEGFP-N1 full-length importin β1; see Method) with a SNAP-tag to replace the fluorescent protein part with the sequence coding for SNAP. After that, cells were transfected and labeled with 1 nM SNAP-tag JF549 dye. The labeling of importin α and β1 with 1 nM SNAP-tag JF549 was sufficient to track individual importin molecules. First, we quantified the number of importins α and β1 inside the NPCs (<50 nm), near NPCs (50–100 nm), or outside NPCs (100–200 nm) [Figs. 6(a) and 6(b)] in live U2OS cells. By employing the two-color MINFLUX imaging strategy, we scanned an ROI to identify an NPC (halo-tag NUP96) and then found the fluorescent importin (SNAP-tag importin α or β1) at different locations of the NPC. By quantifying the fluorescence signal from this scan, we estimated the number of importins α and β1 at different NPC regions (<50, 50–100, and 100–200 nm) in an NPC [Figs. 6(b)–6(d)]. Analyses from two-color MINFLUX images showed that under the same experimental condition, applied bead stress increased the numbers of importin α inside (<50 nm) and near (50–100 nm) NPCs by 65.05% and 100%, respectively, 5-min post stress. Importin β1 increased by 74% and 183%, respectively, 5-min post stress. In contrast, their numbers outside the NPCs (100–200 nm) decreased with stress by 37% and 40% for importins α and β1, respectively [Figs. 6(b)–6(d) and supplementary material Figs. S6(A) and S6(B)]. Similar levels of changes in the number of fluorescence peaks of single-molecule SNAP-tag importin α and β1 were observed 30 min post-stress [Figs. 6(c) and 6(d)]. Sixty minutes after stress cessation, the numbers of importin α and β1 inside and near NPCs returned to the prior-stress levels [Figs. 6(c) and 6(d)]. However, the number of importins β1 outside NPCs remained lower than the prior-stress level [Figs. 6(c) and 6(d), and supplementary material Figs. S6(A) and S6(B)]. For hyper-osmotic stress (410 mOsm), the number of SNAP importins α and β1 decreased inside (<50 nm) and near NPC (50–100 nm) location at 5- or 30-min post-hyper-osmotic stress [supplementary material Figs. S6(C) and S6(D)]. The importins returned to the baseline level of 60 min post-hyperosmotic stress for importin β1 but not for importin α [supplementary material Figs. S6(C) and S6(D)].

**FIG. 6.**
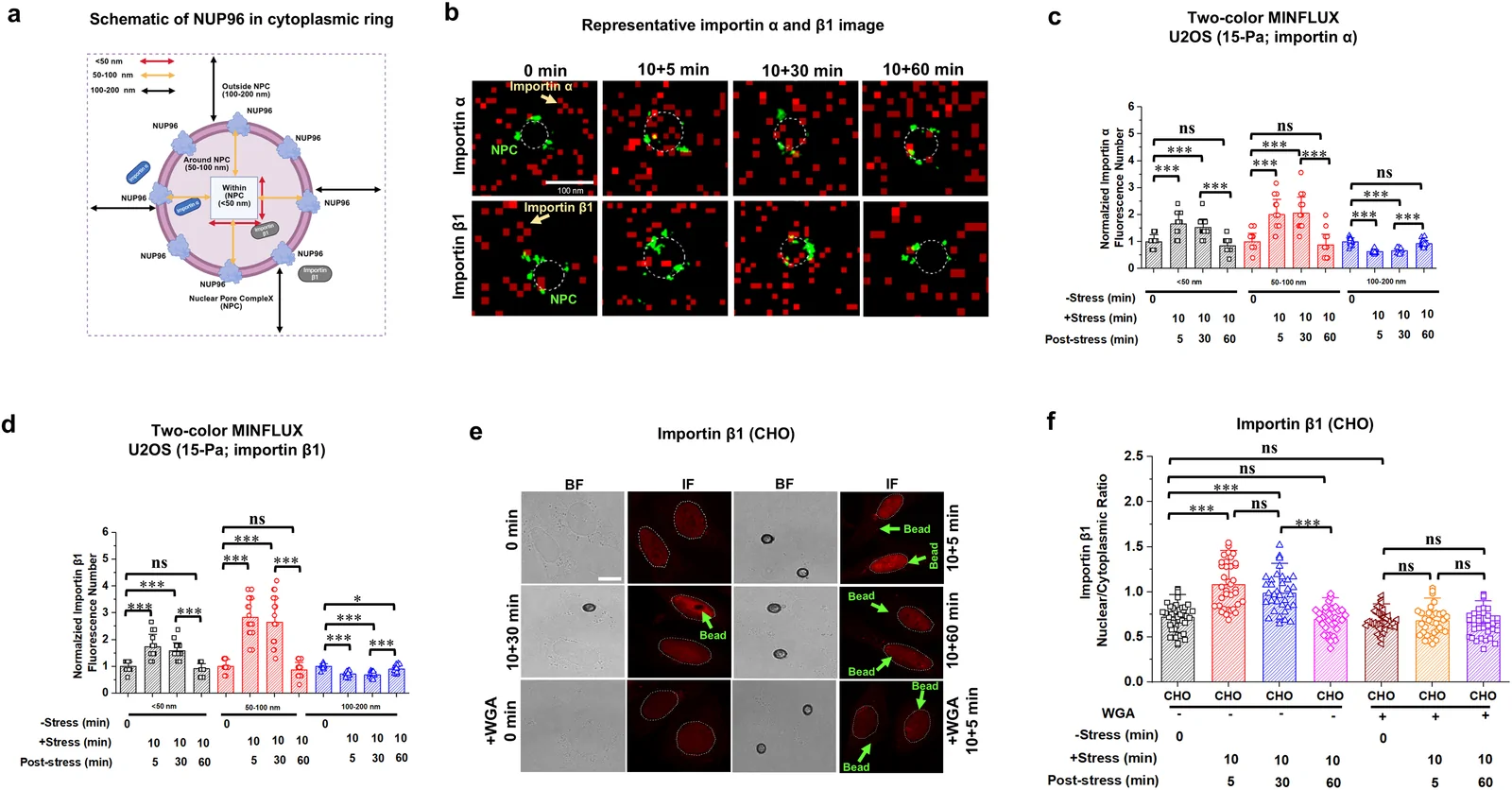
Local cyclic stress induces nuclear importin α and importin β1 translocation. (a) Schematic of various locations of importin α and β1, within (<50 nm) NPC, around (50–100 nm), and outside NPC (100–200 nm) to quantify fluorescence peak of SNAP-Tag importin α or importin β1 (labeled with JF549) quantified by two-color MINFLUX in live U2OS cells. (b) Representative SNAP-tag importin α and β1 images at different locations within and outside of SaraFluor 650B tagged NPC in U2OS cells at 0 min (no stress; control), 5-, 30-, or 60-min after stress, quantified via two-color MINFLUX. Scale bar, 100 nm. The white dashed line represents NPC location in U2OS cells. Individual red dot signal represents a single molecule of SNAP-tag importin α or β1 stained with JF549 dye. (c) Normalized number of single-molecule importin α at no stress, 5-, 30-, or 60-min after 10-min stress of 15 Pa at 0.3 Hz at various locations within, around, and outside the NPC. n = 19 NPCs at no stress; n = 18, 19, and 19 NPCs 5-, 30-, and 60-min after stress from three independent experiments. (d) Normalized number of single-molecule importin β1 at no stress, 5-, 30-, or 60-min after stress at different NPC locations (<50, 50–100, and 100–200 nm). n = 21 NPCs at 0 min; n = 20, 19, 20 NPCs 5-, 30-, and 60-min after stress; three independent experiments. (e) Representative brightfield (BF) and immunofluorescence (IF) of importin β1 in CHO cells at no stress, 5-, 30-, or 60- min, or WGA (50 *μ*g ml^−1^ for 30 min)-treated cells without stress or 5 min after 10-min stress of 15 Pa at 0.3 Hz. Scale bar, 10 *μ*m. (f) Nuclear to cytoplasmic ratio of quantified importin β1 in CHO cells at no stress, and 5-, 30-, or 60-min after 10-min stress, or WGA-treated cells at 0-, 5-, or 60-min after 10-min stress applied via RGD-coated 4-*μ*m ferromagnetic bead attached to the cell apical surface. n = 41, 36, 35, 36 cells at no stress, 5-, 30-, and 60-min after stress; n = 42, 35, and 35 cells at no stress, 5- and 60-min after stress for WGA-treated cells; three independent biological experiments. ^*^P < 0.05, ^**^P < 0.01, ^***^P < 0.001; ns, not statistically different. All P values were quantified using One-way ANOVA with Tukey's test and the Mann–Whitney test.

To confirm these findings of stress-induced nuclear translocation of importins from the two-color MINFLUX, we next performed an immunofluorescence stain for endogenous importin β1 in CHO cells [Fig. 6(e)]. The nuclear–cytoplasmic ratio of importin β1 at various time points after local stress application was quantified from immunofluorescence staining [Fig. 6(f)]. The nuclear to cytoplasmic ratio of importin β1 was quantified for cells at 0 min (no stress; control) [Figs. 6(e) and 6(f)]. After 10-min bead stress, the ratio of the nuclear to cytoplasmic importin β1 in CHO cells was elevated 5- and 30-min post-stress but returned to the baseline level 60-min after stress cessation [Figs. 6(e) and 6(f)]. WGA-treatment, which is a known NPC blocker, completely abolished stress-induced nuclear translocation of importin β1 [Figs. 6(e) and 6(f)]. Additional experiments demonstrate WGA treatment does not block stress-induced FRET efficiency change and NPC dilation in CHO cells (supplementary material Fig. S7). This suggests that WGA does not prevent NPC dilation but only blocks transport through the NPC. Data from both two-color MINFLUX and endogenous staining of the nuclear to cytoplasmic ratio of importins suggest that mechanomemory of NPC dilation increases the nuclear translocation of importins α and β1. In contrast, NPC constriction reduces nuclear translocation of importin α and β1.

### NPC dilation promotes importin β1-dependent HDAC3 translocation into the nucleus

Next, we examined the potential significance of stress-induced NPC dilation. It had been reported that in response to applied forces, HDAC3 shuttles from the cytoplasm into the nucleus to condense the chromatin to regulate gene expression.^51^ To track HDAC3 nuclear translocation and identify the functional impact of NPC dilation, U2OS cells were transfected with HDAC3-tGFP; the nuclear-to-cytoplasmic HDAC3 ratio was dynamically tracked and quantified at 0-, 5-, 30-, and 60-min after stress in the same live cell. HDAC3 nuclear translocation increased 5- and 30-min after stress cessation and returned to baseline at 60-min after stress cessation [Figs. 7(a) and 7(b)]. Similarly, after a 10-min bead stress, the ratio of the nuclear to cytoplasmic HDAC3 in CHO cells was elevated at 5- and 30-min post-stress in fixed cells quantified using immunofluorescence [Figs. 7(c) and 7(d)]. This suggests that memory HDAC3 translocation occurs in different cell types. Blocking NPC-mediated transport by using WGA (50 *μ*g ml^−1^ for 30 min) in the digitonin-containing permeabilization buffer abolished stress-induced HDAC3 nuclear translocation in both U2OS and CHO cells [Figs. 7(a)–7(d)].

**FIG. 7.**
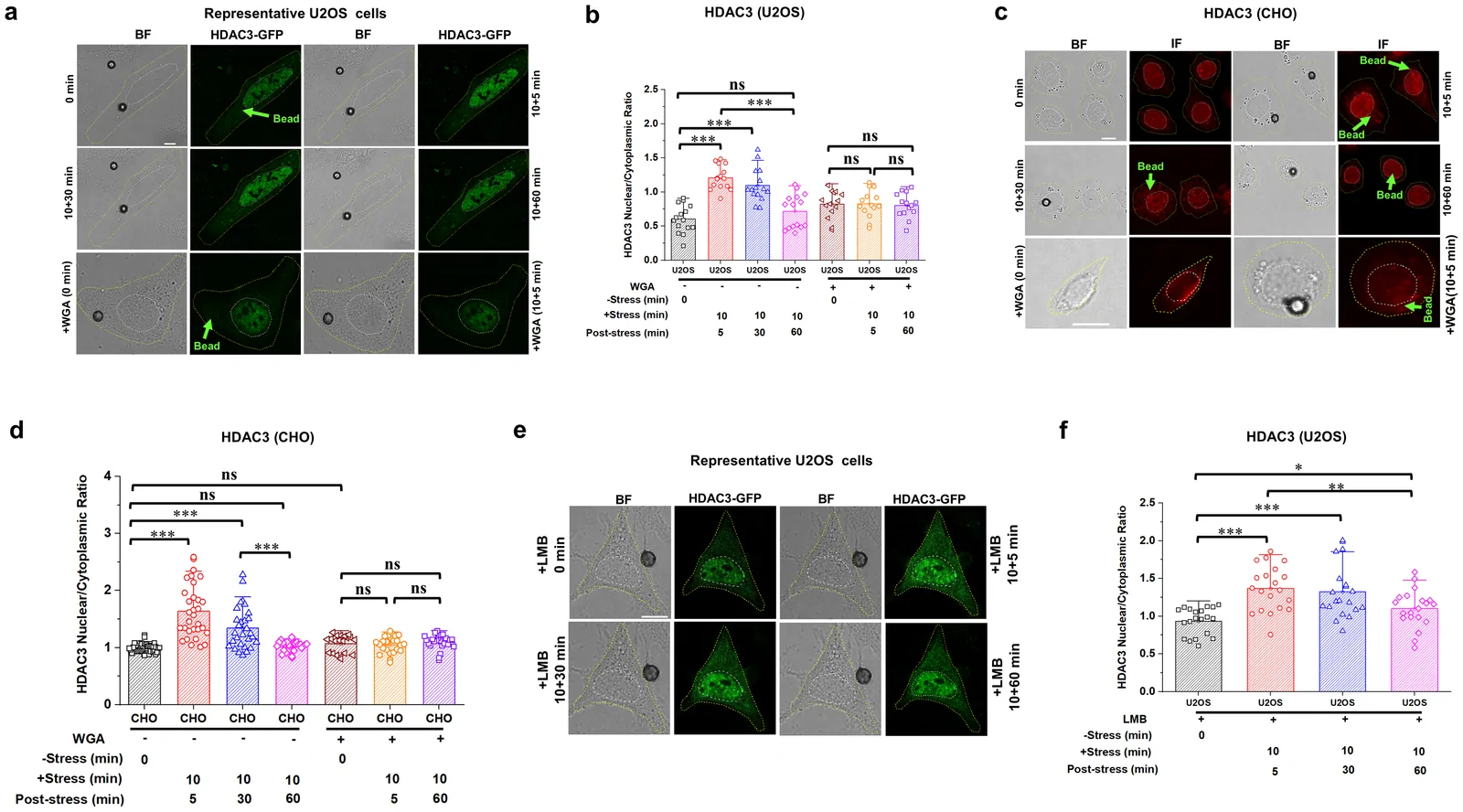
Stress induces HDAC3 nuclear translocation via NPC dilation. (a) Representative images of brightfield (BF) and live HDAC3-GFP (tGFP-tagged HDAC3) transfected U20S cells at no stress, and 5-, 30-, or 60-min after 10-min stress of 15 Pa at 0.3 Hz or WGA (50 *μ*g ml^−1^ for 30 min)-treated cells with 0- and 5-min after 10-min stress. Scale bar, 10 *μ*m. White and yellow dashed lines represent the nuclear envelope and the plasma membrane, respectively, for (a), (c), and (e). (b) Summarized data of the nuclear to cytoplasmic ratio from (a). n = 15 cells at no stress, 5-, 30-, or 60- min after stress, and n = 14 cells at no stress, 5-min, or 60-min after stress for WGA-treated cells; from three independent experiments. (c) Representative images of brightfield (BF) and immunofluorescence (IF) of CHO cells at no stress and 5-, 30-, 60-min, or WGA-treated cells with no stress and 5 min after 10-min stress of 15 Pa at 0.3 Hz. Scale bars, 10 *μ*m. (d) Summarized data of the nuclear to cytoplasmic ratio from (c). n = 43, 31, 29, and 27 cells, respectively, at no stress, 5-, 30-, and 60-min after 10-min stress without WGA. n = 20, 25, and 25 cells, respectively, at no stress, 5-, and 60-min after 10-min stress in WGA-treated cells; three independent experiments. (e) Live HDAC3-tGFP-transfected U20S cells without or with Leptomycin B (LMB) pretreatment (100 nM for 60 min). Representative images of brightfield (BF) and live HDAC3-GFP images at no stress, 5-, 30-, or 60-min after 10-min stress of 15 Pa at 0.3 Hz. Scale bar, 10 *μ*m. (f) Summarized data from cells in (e). n = 20 cells at no stress, 5-, 30-, or 60-min after stress, from three independent experiments. ^*^P < 0.05, ^**^P < 0.01, ^***^P < 0.001; ns, not statistically different. All P values were quantified using One-way ANOVA with Tukey's test and the Mann–Whitney test.

To identify the impact of nuclear export on stress-induced mechanomemory of HDAC3 nuclear translocation, cells were treated with Leptomycin B (LMB) (100 nM for 1 h), a known blocker of CRM1-dependent nuclear export.^52–54^ Stress elevated HDAC3 nuclear translocation 5- and 30-min after stress in LMB-treated cells [Figs. 7(e) and 7(f)]. The percentage increase after LMB treatment (∼40% to 46%) [Fig. 7(f)] is much less than that in untreated cells (80%–100%) [Fig. 7(b)] at 5- and 30-min post stress. These data suggest that export is an active mechanism that is still operable despite physical dilation at 5- and 30-min post stress. Interestingly, this elevation in HDAC3 nuclear translocation stays elevated even 60 min after stress cessation [Figs. 7(f) and 7(g)]. These results suggest that blocking nuclear export allows more accumulation of HDAC3 inside the nucleus, which could explain the longer duration of mechanomemory after bead stress cessation.

Next, we focus on identifying the mechanism for stress-induced HDAC3 nuclear translocation. It is known that nuclear localization of HDAC3 depends on a nuclear localization signal that binds directly to importin β1.^55^ Hence, we hypothesized that HDAC3 nuclear translocation depends on its binding to importin β1. To test our hypothesis, U2OS cells were treated for 2 h with 50 *μ*M Ibetazol, a known specific inhibitor of importin β1.^56^ Stress-induced nuclear translocation of HDAC3 was completely abolished in Ibetazol-treated cells, and as a result, there was no difference between the nucleocytoplasmic ratio of HDAC3 prior-stress and that of post-stress [supplementary material Figs. S8(A) and S8(B)]. These results suggest that importin β1 nuclear translocation was necessary for stress-induced nuclear translocation of HDAC3. Together, these data suggest that the local stress-induced NPC dilation memory prolongs the translocation of importin β1 that facilitates HDAC3 nuclear localization.

## DISCUSSION

In the current study, we show that externally applied physiologically relevant local and global stresses to the surface of living cells induce mechanomemory of deformation of NUPs in NPCs. We reveal that following local cyclic shear stress or global hypo-osmotic stress, the separation distances between nearby NUPs within a single NPC elevate and remain elevated for tens of minutes, accompanied by the memory of NPC dilation. In contrast, global hyperosmotic stress induces mechanomemory of NUP distance reduction and reduces NPC size. These results suggest that mechanical loading on individual NUPs to induce local strains on individual proteins is turned into deformation of the large macromolecular NUP assembly of an NPC.

Published work has demonstrated that forces applied to integrin receptors on the cell surface propagate from the tensed cytoskeleton to the LINC complex to stretch chromatin and induce rapid gene transcription.^57,58^ Because the LINC complex is tethered to the nuclear envelope, it suggests that some of the forces on the LINC are likely to be transmitted to the nuclear envelope, which, in turn, can transmit the stress to the NPCs. The bidirectional response to hypo- and hyperosmotic global stress indicates that NPCs can sustain both tensile and compressive stresses. The persistence of the deformed states of the NPCs after stress cessation suggests that the response is not simply an elastic deformation or viscoelastic deformation but may involve transient plastic deformation, followed by slower structural remodeling, rearrangements, and/or mechanically stabilized states within the NPC. Thus, the observed NUP deformation memory in NUP88 and NUP62 provides a potential molecular basis for mechanomemory of NPCs.

Our current parsimonious working model is that external local stress via integrins leads to nuclear envelope deformation via the LINC plus the nuclear lamina and that global extracellular osmotic stress results in pressure difference across the nuclear envelope via macromolecular concentration differences between the cytoplasm and the nucleoplasm.^59^ The nuclear envelope deformation and the nuclear lamina deformation, in turn, result in NUP deformation and NPC dilation, which facilitates importin entry, followed by HDAC3 nuclear accumulation. However, the present experimental results have only established correlation rather than direct causation between some steps. For example, NPC dilation may correlate with increased transport without being the sole determinant; additional factors including FG-network remodeling or altered transport receptor dynamics may contribute to the elevated HDAC3 nuclear accumulation.

One potential downstream consequence of mechanical deformation is remodeling of the FG-repeat network that forms the selective permeability barrier of the NPC.^11,12^ Because FG-repeat domains are highly dynamic and occupy the central transport channel, changes in NPC geometry or in the relative positioning of FG-Nups could alter the spatial organization, density, or conformational state of the FG network. The NPC dilation rate and importin translocation rate are two different physical parameters. The NPC is a large multi-protein structural assembly, and the rate of dilation/constriction of the NPC depends on the rate of mechanical loading and the NPC's viscoelasticity and plasticity. Importin, on the other hand, is a diffusive molecule, and its rate of translocation depends on many factors: its diffusion rate and its interaction with the FG network, as well as the spatial organization of the FG network. A theoretical modeling report shows that dilation in pore diameter alters the density and mesh size of the FG-Nup network, thereby exponentially enhancing molecular transport rates.^11^ This modeling study shows why relatively small changes in pore geometry can substantially alter nucleocytoplasmic transport of importins. In contrast, because exportins are already highly optimized to interact with FG-Nups, they pass through the NPC efficiently even when the pore is constricted; widening the pore only provides a minor boost to their rate rather than an exponential spike.^11^ These findings provide a plausible explanation of the current experimental observations of stress-induced elevation in importins α and β1 nuclear translocation. A recent report reveals that after mitosis, hydrophobic FG-Nups that form the central transport channel dilate the assembling nuclear pore.^60^ Although our current work does not quantify changes in FG-repeat organization, the persistent dynamic upregulation in NPC diameters provides a structural basis through which mechanical stress influences the FG-Nup barrier remodeling to impact nucleocytoplasmic transport. What might account for the NPC memory effect of the applied stress? We have shown in this study that within 60 s of continuous applied stress, the cells' NUPs exhibit viscoelastic behavior but no memory. If the stress duration is >60 s, the NUPs start to exhibit “short-term plasticity” like memory (lasting over 30 min), which then returns to the prior stress baseline levels. However, currently, we do not know the molecular basis of this “short-term plasticity” observed on the nucleoporins within the NPC. Future work is needed to reveal the molecular mechanisms of time-dependent alterations of the intermolecular bonds between nucleoporins that are responsible for the short-term plasticity.

In this study, we reveal that the mechanomemory of NPC dilation enhances translocation of importin α and β1 through the NPCs, and translocation of importin β1-dependent HDAC3 into the nucleus. Blocking CRM1-dependent nuclear export extends the mechanomemory duration of stress-induced HDAC3 nuclear accumulation. Why stress-induced NPC dilation improves importin specific translocation, but not passive entry, is of particular interest. It has been shown that nuclear force increases permeability across nuclear pore complexes, with a dependence on molecular weight that is stronger for passive diffusion than active transport.^13^ However, for cargos of moderate size and affinity for importins, the FG-Nups barrier is the dominant hurdle. These cargos' active transport could be facilitated by stress-induced elevated rates of importin shuttling across the barrier, although their localization might be driven by passive diffusion under low-tension conditions (i.e., no external stress). HDAC3, with a molecular weight of 49 kDa, is a moderate-sized cargo. As such, after local stress stimulation, its nuclear accumulation may primarily reflect the action of importin β1 dependent active transport but not passive entry of HDAC3.

Our current results are consistent with the published report using cryo-electron tomography (CryoET) to show that NPCs constrict under conditions of energy depletion or hyper-osmotic shock.^17^ In contrast, a recent study using STORM (Stochastic Optical Reconstruction Microscopy) does not observe NPC diameter alteration under hypotonic or hypertonic conditions or ATP depletion in permeabilized U2OS cells.^61^ The exact nature of this discrepancy is unclear, but it may be partly due to the spatial resolution of STORM of ∼13 nm, not as good as CryoET and MINFLUX. Nevertheless, our current study of NPC size mechanomemory and importin and HDAC3 transport memory extends the published findings from osmotic challenges to local dynamic stress applied to integrins in living intact cells on various elastic and viscoelastic substrates.

In general, FRET relies on free fluorophore rotation, and FRET distance calculations assume dynamic isotropicity in which the orientation factor κ^2^ equals 2/3 [the Förster distance R_0_ is proportional to (κ^2^)^1/6^]. This requires that donor and acceptor fluorophores rotate freely on a timescale faster than the fluorescence lifetime, but this assumption may not be valid in the crowded environment of the NPC. For this reason, we interpret our calculated changes in distances as approximate chromophore–chromophore separations rather than exact absolute backbone distances of protein structures. Consequently, we consider decreasing FRET as increasing the separation distance between donors and acceptors (CFP-NUP88 and YFP-NUP62), which is an indication of NUP stretching. In contrast, increasing FRET indicates a decrease in the separation distance between donors and acceptors (CFP-NUP88 and YFP-NUP62), which is an indication of NUP compression. While FRET changes may indicate changes in donor–acceptor separation, FRET changes can also be influenced by fluorophore orientation, local protein rearrangements, and conformational changes of the proteins. Our calculations of strains (deformation) on two different nucleoporins, CFP-NUP88 and YFP-NUP62, in the crowded NPC rely primarily on relative changes in FRET efficiency between conditions, which are less sensitive to uncertainties in κ^2^ if fluorophore rotational constraints remain comparable across experimental conditions. This same strategy was successfully employed in one of our earlier studies, where direct dissociation of the CFP-SMN and YFP-coilin complex in a crowded Cajal body is observed in response to local magnetic bead stress on the cell surface.^37^ Nevertheless, independent MINFLUX measurements of NPC strain memory following the same local mechanical or global osmotic stress mirror the magnitude of NUP strain memory in the NPC observed via FRET. This cross-method validation supports the interpretation that the decreased FRET ratio primarily represents NUP stretching.

FRET is a unique and sensitive method that can be used to report local stress-induced dynamic nucleoporin deformation during magnetic bead stress applications, but FRET only reports relative nucleoporin deformation (stretching vs compressing), not absolute deformation on a nanometer scale. In contrast, MINFLUX reports structural information of proteins at ∼3 nm in spatial resolution.^22^ While the technical advantages of MINFLUX over existing microscopy and the power of MINFLUX for high spatiotemporal precision 3D tracking of diffusing molecules have been reported in detail recently in permeabilized cells.^23^ MINFLUX NPC imaging and tracking of single-molecule importin α and β1 have not been reported before for intact living cells. We report in this study the successful implementation of two-color MINFLUX in intact living human cells. However, image visualization via MINFLUX is incompatible with simultaneous local stress application via mechanical devices such as magnetic twisting cytometry, AFM, or optical tweezers. Hence, the MINFLUX method cannot report dynamic changes of NPC sizes or track dynamic movements of single molecules like importin α and β1 during local bead stress application in the same living cell. However, it can report these dynamics after local stress cessation. In a similar vein, CryoEM is not compatible with local stress-applying devices and thus cannot be used to quantify dynamic local stress-induced changes in nanostructures in living cells. In the current study, we employed FRET spectroscopy (both intensity-based and lifetime-based) to discover dynamic changes of nucleoporins in nanometer resolution during and after local and global stress application. From FRET experiments, we revealed the mechanomemory of nucleoporins. Then, we used one- or two-color MINFLUX to quantify memory of NPC diameter dilation and of single-molecule importin α and β1 movement after local bead stress and global osmotic stress. FRET spectroscopy and two-color MINFLUX nanoscopy are complementary, and our conclusions are supported by using these two independent methods. However, MINFLUX cannot be used to track the same individual NPCs as a function of time, but FRET measurements can track the same individual nucleoporin pairs with time. This is a major advantage of the FRET assay, which can also track the same NUP pairs in cells plated on substrates of various elasticity and viscoelasticity with time, which is not possible for MINFLUX, CryoEM, CryoET, or other modes of super-resolved nanoscopy.

This study uses magnetic twisting cytometry (MTC) for the application of local stress and an osmotic hypertonic or hypotonic medium for the application of global stress. The MTC local stress of 15-Pa is within the physiological range of tractions generated by living cells of 10–100 Pa. Assuming there is little decay in the peak stress amplitude from the cell surface to the nuclear envelope^62,63^ and the tensile stress at NPC is 10-15-Pa, we estimate that the Young's modulus of an NPC is 77-115 Pa [10-Pa or 15-Pa bead stress divided by the tensile strain of 0.13; see Fig. 5(d)]. However, if the stress concentrates around the magnetic bead edge on the cytoplasmic side of the cell surface^64^ and/or on the nuclear envelope along the stress fibers (prestressed F-actin bundles),^65^ the estimated Young's modulus of the NPC could be higher. The osmotic stress magnitude across the plasma membrane is estimated to be ∼171 kPa for hypotonic stress (changing from 300 to 220 mOsm after adding 30% DI water) and ∼235 kPa for hypertonic stress (changing from 300 to 410 mOsm after adding 0.1 M PEG300). These hypo- and hyper-osmotic stresses are 4 orders of magnitude greater than the local bead stress, but cell volume regulation processes are activated to limit the extent of volume changes. In this study, the tensile strains quantified from changes in NUP distances with FRET or NPC diameter changes with MINFLUX post local stress are similar, suggesting that the applied local stress is potent enough to induce NPC dilation memory. In contrast, high global osmotic stress could introduce substantial secondary effects such as global ion fluxes, cytoskeletal remodeling, and prestress alterations, which, in turn, might alter NPC structure and modulus.^41^ Future studies are needed to determine the mechanisms of dynamic changes in nucleoporin deformation under global osmotic stress.

It will be interesting to extend our current findings to help understand molecular mechanotransduction mechanisms by quantifying structural mechanomemory of other proteins in the nuclear envelope and other nucleoporins in NPCs. Our current results are consistent with the recent finding that the NPC scaffold acts as an annular spring to safeguard the nuclear envelope^20^ and extend it to the short-term plastic deformation after >1 min stress application. Together with our earlier findings on nuclear mechanomemory of protein diffusivity elevation and RNA polymerase II activity,^35,36^ our current findings suggest that the impact of forces on the cell is complex and the forces exert their influences at many compartments and components of the cells simultaneously or with some delays in time. Nuclear mechanobiology is critical to the understanding of cell mechanobiology. Recently, it was proposed that several central questions in nuclear mechanobiology are unresolved.^66^ It remains to be determined how the cell integrates the effects of forces on its subunits via feedback loops of its nuclear deformation^67^ and responds in a concerted manner to maintain its physiological, mechanical, and structural homeostasis.

## METHODS

### Cell culture

In this work, CHO (Chinese hamster ovary) DG44 cells and U2OS-CRISPR-NUP96-Halo cells (Cytion) were used. CHO DG44 cells were grown in Ham's F12 medium with 10% DFBS [dialyzed fetal bovine serum (FBS)] by following the protocol mentioned previously.^35,36,57^ CHO DG44 and Halo-tag U2OS-CRISPR-NUP96 cells were cultured with McCoy's 5a medium (Cytion) supplemented with 10% FBS (fetal bovine serum), 3.0 g/l Glucose, Glutamine, 2.0 mM Sodium pyruvate, and 2.2 g/l NaHCO_3_ by following the manufacturer's protocol. CHO DG44 and U2OS-CRISPR-NUP96-Halo cells were cultured and seeded every 3–4 days using TrypLE Express (Thermo Fisher Scientific) and StemPro™ Accutase™ (STEMCELL Technologies), respectively, and grown in a humidified incubator at 37 °C and 5% CO_2_. For both CHO and U2OS cells, mycoplasma contamination was experimentally monitored visually by staining with DAPI (4′,6-diamidino-2-phenylindole). No mycoplasma contamination was observed during cell culture, growth, and experiments for either of these two cell lines.

### Ferromagnetic bead coating and osmotic stress

RGD peptides were used to coat the 4-*μ*m ferromagnetic (Fe_3_O_4_) beads [obtained from Dr. J. Fredberg's laboratory (Boston, MA)] to bind to integrin cell surface receptors and apply local cyclic stress by using one-dimensional magnetic twisting cytometry (1D-MTC). For this, 1 ml carbonate buffer of pH 9.4 was added to two hundred *μ*l of 4-*μ*m diameter ferromagnetic beads solution. RGD peptides were then added by following the protocol mentioned previously to a final concentration of 50 *μ*g per ml.^68^ RGD peptides were mixed overnight with 4-*μ*m ferromagnetic beads at 4 °C by using a rotator.

For extracellular osmotic pressure, a hyperosmotic (addition of 0.1 M PEG 300) or hypo-osmotic stress (addition of 30% DI water) was used in CHO and U2OScell culture medium.^46,69^ In both hyper- and hypo-osmotic pressure conditions, isotonic cell-culture medium (∼300 mOsm) was considered as a control. Addition of 30% DI water generates a hypotonic pressure of −80 mOsm to swell CHO or U2OS cells to expand NPCs. In contrast, the addition of 0.1 M PEG 300 applied an additional osmotic pressure of +110 mOsm to contract CHO or U2OS cells and NPCs.^46,69^

### Magnetic twisting cytometry (MTC)

MTC works based on magnetizing a ferromagnetic bead in one axis direction and applying a twisting magnetic field in the normal/orthogonal axis direction to generate twisting torque in the plane between these two axes.^65^ In this work, MTC was used to apply local cyclic stress by adding RGD-coated 4-*μ*m ferromagnetic beads to integrin cell surface receptors. For FRET, around 100 000 CHO or U2OS cells were seeded and incubated overnight in an incubator at 37 °C and 5% CO_2_. CHO DG44 cells were grown on a fibronectin-coated (Thermo Fisher Scientific, 5 *μ*g/ml) 35 mm gridded dish (81148, Ibidi USA Incorporated, Madison, WI, 50 *μ*m cell location) to precisely locate the same cell during, before, and after applying external stress. U2OS cells were grown on #1.5 H high-performance 35 mm glass bottom dish (20 mm micro-well). For MINFLUX imaging, 150-nm positively charged (OD50, catalog: CC11-150-POS-DIH-50-1) gold nanoparticles were deposited on the bottom of the dish. Positional reference was obtained from the scattering of nanoparticles on the dish with a similar level of Z-axis height to the bottom of U2OS cells.

In this study, NUP88–NUP62 pairs were selected for their locations in the NUP214–NUP88–NUP62 complex at the cytoplasmic ring of the NPC.^19^ In addition, co-overexpression of NUP62 stabilizes NUP88 to promote tumor growth.^70^ Degrading NUP62 during mitosis arrests NPC assembly and removes the whole central transport channel.^60^ Consequently, for FRET, CHO cells were co-transfected (one single transient transfection with two plasmid DNAs together) with 500 ng of mouse NUP88 (CW310883; monomeric mCFP; OriGene Technologies Inc.) plasmid DNA with 250 ng of NUP62 (CW310884; monomeric mYFP; OriGene Technologies Inc.) for 12 h. For control, cells were also co-transfected with distant NUPs of NUP88 and NU43 (CW311443; monomeric mYFP; OriGene Technologies Inc.), or NUP88 with NUP133 (CW311444; monomeric mYFP; OriGene Technologies, Inc.) and incubated for 12 h in a humidified incubator at 37 °C and 5% CO_2_. U2OS-CRISPR-NUP96 halo-tagged cells were stained for 1 h with haloTag SaraFluor 650B ligand (250 nM; Goryo Chemical). 2 × 10^5^ RGD-coated ∼4 *μ*m ferromagnetic bead solution [30 *μ*l bead solution (1 mg bead/ml cell culture medium)] was added for 20 min on co-transfected CHO DG44 or live-stained U2OS cells to apply local cyclic stress via MTC. To remove unbound beads, cells were washed once with cell culture medium. Finally, 2 ml of fresh cell culture medium was added in 10 mM HEPES buffer for CHO DG44 or FluoroBrite DMEM (ThermoFisher) with 10% FBS (Gibco) for U2OS cells.

For fluorescence lifetime imaging (FLIM)-FRET, CHO DG44 cell dishes were set in a stage-top Okolab incubator (Leica Microsystems), or inside an MTC coil or MINFLUX stage for dynamic FRET imaging. First, 0 min (no stress; control) stress and time control dynamic FRET data were quantified. Simultaneous sequential YFP and CFP emission images of NUP88, NUP62, NUP43, and NUP133 with applied stress (50 G at 0.3 Hz) were acquired using a dual-view (Optical Insights) system integrated with an inverted Leica microscope, as mentioned previously.^37^ For the mechanomemory of NPC dilation experiment, after the 0-min stress intensity FRET and FLIM-FRET experiments, cell dishes were transferred onto an MTC chamber with magnetic field coils. Cells were first magnetized along the Y direction (magnetizing pulse of ∼1000 G; ∼10 ms) and then applied a sinusoidal twisting field of 50 G was applied at 0.3 Hz, along the Z-axis direction. A 50 G applied magnetic field corresponded to 15 Pa shear stress, as mentioned previously and quantified using magnetic moment and bead contact area to cells.^57^ The sinusoidal cyclic stress of 15 Pa at a frequency of 0.3 Hz was applied for 10 min for both cell lines grown on rigid dish (∼2 GPa), elastic, and viscoelastic substrates of 2 and 20 kPa. FRET efficiency, NPC diameter, importin α and β1 tracking, and nuclear HDAC3 translocation were quantified at 5, 30, or 60 min after stress.

### FRET probe and plasmids

For NPCs, plasmids of nucleoporin mCFP-NUP88, mYFP-NUP62, mYFP-NUP43, and mYFP-NUP133 were purchased from OriGene Technologies Inc. The vector includes PS100044 for nucleoporins mCFP NUP88 and PS100042 for NUP62, NUP43, and NUP133 from OriGene Technologies Inc. with pCMV6-AC [Expression promoter: CMV (Cytomegalovirus promoter). The C-terminal tag was used with either mCFP on NUP88 or mYFP on NUP62, NUP43, or NUP133] for mouse NUP88, NUP62, NUP43, and NUP133. These plasmids were used for the co-transfection of CHO DG44 cells using Lipofectamine 3000 (Invitrogen) according to the manufacturer's protocols. Co-transfection with two different plasmids (mCFP-NUP88 and mYFP-NUP62 or mCFP-NUP88 and mYFP-NUP43, or mCFP-NUP88 and mYFP-NUP133) was expressed as C-terminal fluorescent fusion proteins, resulting in three different protein pairs (NUP88-mCFP and NUP62-mYFP; or NUP88-mCFP and NUP43-mYFP; or NUP88-mCFP and NUP133-mYFP) in different cell culture dishes.

For importin α and importin β1 tracking experiments in two-color MINFLUX, pDsRed importinα (Addgene #119679) and pEGFP-N1 full-length (Addgene #106941) vectors were used to subclone SNAP-tag importin α and β1 in U2OS cells. These plasmids were used for transfection in live U2OS cells to make an orthogonal tag to visualize and track importin α and β1.

### Live fluorescence lifetime imaging (FLIM)-FRET

Lifetime (nanoseconds) and emission-based FRET efficiency were quantified to assess the separation distance between nucleoporin donor and acceptor to identify their expansion and contraction. This eventually assesses NPCs for dilation and constriction mechanomemory during and after externally applied local cyclic and global osmotic stress.

For live FRET emission images, a dual-view (OI-04-SEX2) system was used to acquire simultaneous emissions of the donor (CFP-NUP88), or acceptor (YFP-NUP62, YFP-NUP43, or YFP-NUP133). FRET efficiency was quantified using the YFP to CFP ratio of donor and acceptor. This system includes the following filters with CFP (excitation S430/25 and emission S470/30), YFP (excitation S500/20 and emission S535/30). A 515-nm dichroic mirror was used to split the two emission images. For live cell FRET, co-transfected CHO cells were excited at the CFP wavelength, and quantified both YFP and CFP emissions simultaneously on the same output screen using a Hamamatsu CCD camera (C4742-95-12ERG). This system is integrated with MTC, and an inverted Leica microscope with a 63× oil objective (NA 1.32) was used to acquire FRET images.

For lifetime-based FRET images, a scanning mirror module (M612) with a Fast FLIM (M210); ISS from ISS Alba (Champaign, USA) integrated with a Leica DMi8 microscope was used. A pulsed 440/488-nm nanosecond laser with a 50 MHz repetition rate through an apochromatic 63× oil objective (NA 1.40) was used to acquire detected photons in a TTTR (time-tagged-time-resolved) format. To quantify the FRET-lifetime, CHO DG44 cells were grown on a 35 mm gridded dish and co-transfected with mCFP NUP88 (donor) and mYFP NUP62 (acceptor) for 12 h. After adding RGD-coated 4-*μ*m ferromagnetic beads to integrin cell surface receptors, the FRET lifetime (ns) was quantified at 0 min (no stress; control). Magnetic twisting cytometry (MTC) was then used to apply stress (15 Pa at 0.3 Hz) for 10 min and quantify FRET lifetime (ns) at 5 and 60 min after stress. Cells transfected only with donor (mCFP-NUP88) were the control and used to quantify FRET efficiency (η) by using the following equation:^35^

$$\eta =1-\frac{\tau_{\text{da}}}{\tau_{d}},$$ (1)

where $\tau_{da}$ is the lifetime (ns) of dual transient co-transfected CFP NUP88-YFP NUP62 cells, and $\tau_{d}$ is the lifetime (ns) of transiently transfected CFP-NUP88 CHO DG44 cells. This quantified FRET efficiency was used to estimate the apparent donor-acceptor distance changes by using the following equation:

$$\eta =\frac{1}{1+{\left(\frac{R}{R_{0}}\right)}^{6}},$$ (2)

η is the quantified FRET efficiency, R is the apparent donor-to-acceptor separation distance, and R_0_ is the Förster distance of this donor–acceptor pair (4.9 nm for CFP-YFP).^44,45^

### Polyacrylamide (PA) elastic substrate fabrication

CHO DG44 cells were grown on fibronectin-coated (30 mg per ml) 2- or 20-kPa PA (polyacrylamide) gel substrates to quantify the effect of elastic substrate stiffness on mechanomemory of NPC dilation. PA gel substrates were made on a 35-mm gridded glass-bottomed dish (Catalog #81148, Madison, WI, Ibidi USA Incorporated) by following the previously published protocol.^36,71^ In brief, 2- or 20-kPa elastic PA substrates were made by varying mixing concentrations of 2% bis-acrylamide (Cat#161-0142; Bio-Rad) and 40% acrylamide (Catalog#1610140; Bio-Rad). A 0.33 or 0.934 ml of 2% bis-acrylamide and 1.25 or 2.5 ml of 40% acrylamide were added, respectively, in water to make a final volume of 10 ml for 2- or 20-kPa PA substrates.

On PA elastic substrates, 100 000 CHO DG44 cells were grown and incubated overnight in an incubator at 37 °C and 5% CO_2_. After that, cells were co-transfected with CFP NUP88 and YFP-NUP62 plasmid DNA for 12 h in a humidified incubator at 37 °C and 5% CO_2_. RGD-coated 4-*μ*m ferromagnetic beads were then added, and FRET images were acquired at 0 min (no stress; control). FRET efficiency at 0 min stress was first quantified and compared for 2 and 20 kPa PA substrates. A sinusoidal cyclic stress of 15 Pa at 0.3 Hz was then applied for 10 min, and FRET images were acquired 5-, 30-, and 60-min after stress. FRET efficiency at 5-, 30-, and 60-min after 10 min stress was quantified to identify the mechanomemory of NPC dilation after stress cessation on 2- or 20-kPa PA elastic substrates.

### Viscoelastic substrates

Mechanomemory of NPC dilation was quantified for slow (∼1000 s) and fast (∼200 s) relaxing viscoelastic substrates of 2 and 20-kPa. All slow- and fast-relaxing viscoelastic substrates were fabricated by following the previously published protocol.^72,73^ Briefly, slow-relaxing 2- or 20-kPa viscoelastic substrates were made by mixing sodium alginate (Catalog#42000101, molecular weight >200 kDa and viscosity >200 mPa s; Sigma–Aldrich) and calcium sulfate solutions. For both these two viscoelastic substrates, an oligopeptide GGGGRGDSP (Peptide International) was used to make a cell-adhesive surface by following the protocol previously published.^73,74^ For this, 1 g alginate was reconstituted [1% w/v in MES buffer (0.1 M MES, 0.3 M NaOH, pH 6.5)], followed by the addition of N-(3-dimethylaminopropyl)-*Nʹ*-ethylcarbodiimide hydrochloride (EDC; Catalog#800907; Sigma-Aldrich) at a ratio of 1:20 to the monomers of uronic acid in alginate. After that, N-hydroxysulphosuccinimide (Sulfo-NHS; Catalog#24510, Thermofisher Scientific) was added at a molar ratio of 1:2 to EDC and 150 *μ*M of peptide sequence to make a 2 oligopeptide on average per alginate polymer. This complete reaction was stirred for 20 h, followed by dialysis against DI water for three days with 0.5 g of activated charcoal. A 0.22-*μ*m sterilized filter was used for filtration and to dry and freeze these modified alginate viscoelastic substrates. The coupling efficiency between alginate and oligopeptide was characterized before by using 125I-labeled oligopeptide.^75^ These modified sodium alginates were dissolved in DMEM (Catalog# 11-965-092, Thermo Fisher Scientific) at 2.5% (wt./vol.) stock solution, and calcium sulfate was used as a crosslinker (35 and 180 mM) for the fabrication of slow-relaxing viscoelastic substrates.

For fast-relaxing, high-viscosity sodium alginate (Catalog#42000101, molecular weight >200 kDa and viscosity >200 mPa s; Sigma–Aldrich) were mixed with low viscosity Sodium alginate (Catalog#42000501, molecular weight <75 kDa and viscosity <20 mPa s; Sigma–Aldrich) at a ratio of 2:1. 55 and 300 mM calcium sulfate were used to cross-link fast-relaxing 2- or 20-kPa viscoelastic substrates. Before growing cells, viscoelastic substrates were washed to remove unbound crosslinkers. After that, 100 000 CHO DG44 cells were grown and incubated overnight in an incubator at 37 °C and 5% CO_2_. Cells were co-transfected with CFP NUP88 and YFP NUP62 for 12 h in a humidified incubator at 37 °C and 5% CO_2_. After adding 4-*μ*m RGD-coated ferromagnetic beads, FRET images were taken for 0 min (no stress; control). A sinusoidal cyclic stress of 15 Pa at 0.3 Hz was then applied for 10 min, and FRET images were collected for 5-, 30-, and 60-min after stress. FRET efficiency at 5-, 30-, and 60-min after 10-min stress was quantified to identify the mechanomemory of NPC dilation on slow- and fast-relaxing viscoelastic substrates.

### Protein labeling and live cell MINFLUX imaging

U2OS-CRISPR-NUP96 halo-tagged cells were used to quantify the mechanomemory for the nominal diameter of the NPCs. An Abberior instrument of MINFLUX microscope with 561- and 642-nm excitation and 405-nm (continuous wave) activation lasers was used for NPC scaffold and importin α and β1 tracking experiments. In all MINFLUX imaging, a 100× oil-immersion objective with an NA of 1.4 was used. All MINFLUX experiments were controlled by Abberior Imspector software (v16.3.21317-m2410). To minimize drift for both NPC scaffold and importin α and β1 tracking experiments, 150 nm positively charged (OD50, catalog: CC11-150-POS-DIH-50-1) gold nanoparticles were used. For all experiments, drift was minimized with the MINFLUX built-in stabilization system and maintained at less than 0.8 nm in XYZ. All MINFLUX scaffold imaging was performed at the basal nuclear envelope of the cell nucleus and stabilized by using these gold nanoparticles.

For the NPCs scaffold experiment, 100 000 U2OS-CRISPR-NUP96 halo cells were seeded on a glass-bottom dish deposited with 150 nm positively charged gold nanoparticles. 4-*μ*m RGD-coated ferromagnetic beads were then added for 20 min. After washing unbound beads, HaloTag SaraFluor 650B ligand (250 nM; Goryo Chemical) was used to visualize NPCs in live U2OS cells.^49^ NUP96 is a scaffold nucleoporin and is expressed at the cytoplasmic ring and the nuclear ring (8 pairs at each ring). Depletion experiments reveal its significant role in modulating the interdependence of cytoplasmic and nucleoplasmic rings and the central channel of the NPC.^16^ Additionally, NUP96 was compatible with a halo-tag that allowed us to perform experiments with an orthogonal SNAP-tag to track nucleocytoplasmic translocation of importin α and β1. On the other hand, the ON/OFF cycle of SaraFluor 650B dye was sufficient and suitable for MINFLUX imaging that did not require any redox buffer or photoactivity. We could not obtain the fluorescent tag of SaraFluor 650B dye for NUP88 or NUP62. Considering the self-blinking nature of halo-tag SaraFluor 650B ligand and NUPs' structural location, we selected NUP96 for MINFLUX tracking and imaging. Cells were incubated for 1 h with SaraFluor 650B ligand at 37 °C and 5% CO_2_, followed by three 1 h washes to remove free dyes deposited on the dish to reduce the background. For live cell NPC scaffold imaging, the cell culture medium was replaced with imaging medium [FluoBrite supplemented with 10% fetal bovine serum (FBS; Gibco), and 2% GlutaMAX (Gibco)] by following the previously published protocol.^76^ MINFLUX imaging for 0 min stress (no stress; control) for bead stress or at an isotonic medium (∼300 mOsm) was first acquired. Then, due to the self-blinking nature of SaraFluor 650B ligand, another cell dish was transferred to a magnetic twisting cytometry (MTC) coil to apply 10 min stress of 15 Pa at 0.3 Hz or was subjected to hypo-osmotic (addition of 30% DI water; ∼220 mOsm), or hyper-osmotic (addition of 0.1 M PEG; ∼410 mOsm) for 30 min and acquired MINFLUX images. For mechanomemory experiments, multiple dishes were taken for MINFLUX NPC scaffold images, and experiments were performed at 5-, 30-, and 60-min after 10-min of stress at 15 Pa and 0.3 Hz. Similarly, for hypo- and hyper-osmotic stress, MINFLUX NPC scaffold images were taken in different dishes at 5-, 30-, or 60-min after 30-min of osmotic stress. During imaging experiments, at first, a regular confocal scan was performed to visualize the location of NPCs, and then individual NPCs' MINFLUX images were acquired for live U2OS cells. Live cell NPCs are dynamic, and for this, one or two nearby NPCs (by drawing a 200 × 200 nm^2^ rectangular box in XY to locate NPCs labeled with SaraFluor 650B in the individual pore) were scanned and collected for MINFLUX data. This approach is necessary for successful MINFLUX imaging, which is different from permeabilized or fixed cells with the GLOX buffer experiment. For each living NPC, about 150 localization and 50–75 individual trace locations were acquired within 10 min. When we scanned a large region of interest, a gold nanoparticle deposited outside the cell membrane was used as a MINFLUX stabilization reference. Each individual live NPC image was taken from the halo-tag SaraFluor 650B ligand MINFLUX experiment, which identified an eightfold rotational symmetry of NUP96 nucleoporins. Halo-tag SaraFluor 650B ligands were bound to individual nuclear pore scaffolds. The distance between the two opposite sides of rotational symmetry in the eightfold symmetry was taken to quantify the nominal diameter of NPCs for live cells. Circumferential strain [ε = (2πr − 2πr_0_)/2πr_0_; r and r_0_ are the NPC diameters with and without stress] was quantified from average NPC diameter changes before and after stress.

### Live cell importin α and β1 tracking

For these experiments, 100 000 U2OS cells were grown on a #1.5 H 35 mm glass-bottom dish deposited with 150 nm positively charged gold nanoparticles. After overnight incubation, cells were transfected with 100 ng SNAP-tag importin α (pDsRed-importinα; Addgene #119679 subcloning bundled with SNAP-tag importin α) or β1 (pEGFP-N1 full-length importin β1; Addgene #106941 subcloning bundled with SNAP-tag importin β1) using Lipofectamine 3000 (Invitrogen) according to the manufacturer's protocols for 12 h. After that, 4-*μ*m RGD-coated ferromagnetic beads were added for 20 min to apply stress for mechanomemory.

A two-color MINFLUX imaging system was used to visualize the NPC scaffold first in 2D. A 3D NPC scaffold imaging was used to identify the interaction of NPC with importin α or β1 nuclear translocation. After taking MINFLUX images of NPC scaffolds within 10 min, then the single-molecule importin α or β1 was tracked on the same pore in 3D. In the sample preparation, HaloTag SaraFluor 650B ligand (250 nM; Goryo Chemical) was used on live U2OS cells for 1 h to label NUP96. This was then followed by 1 nM SNAP-tag JF549 (Janelia Research) live cell dye incubation to make an orthogonal tag to track importin α and β1. After that, cells were washed three times, each with 1 h in regular cell culture medium. Finally, imaging medium [FluoBrite supplemented with 10% fetal bovine serum (FBS; Gibco), and 2% GlutaMAX (Gibco)] was added before acquiring 3D importin α and β1 images for 0 min stress (no stress; control) for bead stress, or in isotonic medium (∼300 mOsm). Multiple cell dishes were used for different local and global stress durations due to the self-blinking nature of the SaraFluor 650B ligand. After taking 0 min or isotonic medium data, other cell dishes were transferred to an MTC coil to apply 10 min stress of 15 Pa at 0.3 Hz. After applying hypo- or hyper-osmotic stress for 30 min, MINFLUX NPC scaffold and 3D importin α and β1 tracking images were also collected for living intact cells. For mechanomemory experiments, NPC scaffold and importin α and β1 tracking images were taken in different dishes at 5-, 30-, or 60-min after 10-min of stress (15 Pa at 0.3 Hz). Similarly, MINFLUX imaging was performed on different dishes at 30 min of global stress or 5-, 30-, or 60-min post-stress after switching to isotonic medium.

In MINFLUX imaging, a diagonal scanning approach was used to visualize labeled NUP96 (HaloTag SaraFluor 650B ligand) in live cell nuclear pores, thereby acquiring MINFLUX localizations. After that, another scan was performed to locate stained importin α or β1 (SNAP-tag JF549) within a region of 200 × 200 nm^2^ on the same NPC scaffold. MINFLUX tracking was used to observe locations of importin α and β1 at different NPC regions [inside individual NPCs (<50 nm), near NPCs (50–100 nm), or outside NPCs (100–200 nm)]. 3D mean square displacement (MSD(τ) = ⟨[x(t + τ) − x(t)]^2^ + [y(t + τ) − y(t)]^2^ + [z(t + τ) − z(t)]2⟩) was quantified for 100 ms to quantify movements of single-molecule importin α and β1 after local bead or global osmotic stress. The number of JF549 single-molecule importin α or β1 fluorescence peaks was then quantified at different NPC regions. This measurement was used to assess how externally applied local stress or global osmotic stress affects importin α and β1 nuclear translocation.

### Live cell imaging, immunofluorescence, and data analysis

For live cell HDAC3 nuclear translocation experiments, 100 000 U2OS cells were seeded and incubated overnight on a 35 mm gridded dish. After that, HDAC3-tGFP (Catalog# SKU MG226888; OriGene Technologies Inc.) was used to transfect cells with Lipofectamine 3000 (Invitrogen). After overnight (12 h) incubation, RGD-coated 4-*μ*m ferromagnetic beads were added and incubated for 20 min before applying stress. At first, cells were imaged at 0 min (no stress; control). After that, 10 min of stress (15 Pa at 0.3 Hz) was applied, and imaging was performed for the same cells 5, 30, and 60 min after stress.

For immunofluorescence imaging, 100 000 CHO cells were seeded and incubated overnight on a fibronectin-coated 35 mm gridded dish. After overnight incubation, cells were fixed using 4% paraformaldehyde (Santa Cruz) at room temperature for 10 min for 0 min stress (control). After adding RGD-coated 4-*μ*m ferromagnetic beads, 10 min stress of 15 Pa at 0.3 Hz was applied, and cells were fixed at 5-, 30-, and 60-min post-stress. Cell dishes were then rinsed three times with PBS and permeabilized using 0.3% Triton X-100 (Sigma–Aldrich) for 20 min. After 1× PBS washing, normal donkey serum (5%; Jackson ImmunoResearch Laboratories) was added to cells for about 4 h at room temperature. This was followed by overnight incubation with primary HDAC3 antibody (1:100 v/v in 1% BSA; Cell Signaling; 85057) at 4 °C. For nuclear importin translocation, primary importin β1 antibody (1:100 v/v in 1% BSA; Cell Signaling; 60769) was used. After primary antibody incubation, cells were rinsed five times for 5 min each in 1× PBS. Secondary antibody {1:200 v/v; Abcam, ab150062 [donkey anti-rabbit IgG H&L (Heavy & Light chains), Alexa Fluor 555]} was then added and incubated for about 2 h at 4 °C. Finally, all different cell dishes were rinsed three times with 1× PBS and proceeded for imaging.

For the quantification of fluorescence intensity for HDAC3 and importin β1, the integrated density of the cytoplasm and nucleus was quantified after subtracting background intensity by following the previously published method.^35,57^ The ratio of nuclear to cytoplasmic background-corrected integrated density of fluorescence for 0-, 5-, 30-, and 60-min after 10-min stress was taken as the level of immunofluorescence measurement for no stress (0 min; control), and bead stress for both CHO and U2OS cells.

For Lamin A/C staining, 100 000 CHO cells were first seeded and incubated overnight on a fibronectin-coated 35 mm gridded dish. The following day, cells were co-transfected with CFP-NUP88 and YFP-NUP62 and incubated 12 h. After that, cells were fixed with 4% paraformaldehyde (Santa Cruz), permeabilized with 0.3% Triton X-100, and incubated with Lamin A/C Mouse Monoclonal Antibody (Cell Signaling; 4777). Finally, after secondary antibody incubation, immunofluorescence images of Hoechst nuclear stain, CFP-NUP88, YFP-NUP62, and Lamin A/C were taken to identify the NUPs' location in the co-transfected cells.

### Blocking nuclear pore complexes and nuclear stain assays

To block NPC-mediated nuclear transport, WGA was used at a concentration of 50 *μ*g ml^−1^ for 30 min in a permeabilization and transport buffer [digitonin (20 *μ*g/ml), 1 mM ethylene glycol-bis(2-aminoethylether)-N,N,Nʹ,Nʹ-tetraacetic acid (EGTA), 20 mM HEPES, 5 mM sodium acetate, 1 *μ*g/ml aprotinin, 110 mM potassium acetate, 1 *μ*g/ml leupeptin, 2 mM DL-Dithiothreitol (DTT), 1 *μ*g/ml pepstatin, 250 *μ*M adenosine triphosphate (ATP) at pH 7.0]. All these permeabilization buffer chemicals were purchased from Sigma–Aldrich. To verify the location of CFP or YFP nucleoporins and the blocking of NPC-mediated transport, Alexa Fluor 594-conjugated WGA (Catalog#W11262; Thermofisher Scientific) was used. Hoechst (Catalog#33342; Thermofisher Scientific) was used for nuclear staining and overlay images for NUP88 and NUP62 co-transfected live cells or with WGA-treated permeabilized cells.

### Blocking importin β1 nuclear translocation and nuclear export

U2OS cells were treated with 50 *μ*M Ibetazol (Catalog# HY-170316, MedChem Express)^56^ for 2 h to quantify the effect of inhibiting importin β1 on stress-induced HDAC3 nuclear translocation. Briefly, after adding RGD-coated 4-*μ*m ferromagnetic beads, cells were treated with 50 *μ*M Ibetazol for 2 h. After that, cells were fixed at 0 min stress (no stress, control), and 5, 30, or 60 min after 10-min stress of 15 Pa at 0.3 Hz. Cells were then incubated with primary HDAC3 antibody (1:100 v/v in 1% BSA; Cell Signaling; 85057) and secondary donkey anti-rabbit IgG H&L (Heavy & Light chains), Alexa Fluor 555 antibodies. Immunofluorescence images were taken to quantify the nuclear-to-cytoplasmic ratio at 0, 5, 30, or 60 min after 10-min bead stress.

Next, to block CRM1-mediated nuclear export and to quantify stress-induced HDAC3 nuclear translocation in live U2OS cells, 100 000 cells were seeded and incubated overnight on a 35 mm gridded dish. After overnight incubation, HDAC3-tGFP was used to transfect cells by following the manufacturer's protocol. The following day, RGD-coated 4-*μ*m ferromagnetic beads were added. After that, 100 nM Leptomycin B (LMB) (Catalog#HY-16909; MedChem Express) was added for 1 h to block CRM1-mediated nuclear HDAC3 export in live U2OS cells.^52–54^ At first, HDAC3-tGFP imaging was performed at 0 min (no stress; control). After that, 10-min stress was applied, and imaging was performed at 5-, 30-, and 60-min after stress cessation by tracking the same cells on the gridded dish in live U2OS cells.

### MINFLUX data analysis and live cell imaging

For MINFLUX and importin tracking images, MINFLUX-BASE Imspector (v16.3.21317-m2410) and ParaView (5.12.1) software was used to export and analyze data.^76^ A custom-made MATLAB code was used to export the localization, EFO, and CFR data from Imspector (v16.3.21317-m2410) software. In MINFLUX localization imaging, the lower limit of effective frequency at offset (EFO) was set to 25 kHz and the upper limit to 80 kHz to separate NPC localization data from background. This lower limit of EFO values distinguishes NPC localization data of NUP96 (labeled with HaloTag SaraFluor 650B ligand) from background noise. For both NPC scaffold and tracking experiments, a CFR value of 0.8 was used to filter the raw MINFLUX data. For all exported Z-localization tracking data, a correction factor of 0.7 was multiplied for a 100× oil immersion (NA 1.4) objective and cell culture medium in live cells by following the manufacturer's protocol.

## SUPPLEMENTARY MATERIAL

See the supplementary material (Figs. S1–S8) for additional information.

## Data Availability

The data that support the findings of this study are available from the corresponding author upon reasonable request and are also available within the article and its supplementary material.
